# Leveraging Data Locality in Quantum Convolutional Classifiers

**DOI:** 10.3390/e26060461

**Published:** 2024-05-28

**Authors:** Mingyoung Jeng, Alvir Nobel, Vinayak Jha, David Levy, Dylan Kneidel, Manu Chaudhary, Ishraq Islam, Audrey Facer, Manish Singh, Evan Baumgartner, Eade Vanderhoof, Abina Arshad, Esam El-Araby

**Affiliations:** Department of Electrical Engineering and Computer Science, University of Kansas, Lawrence, KS 66045, USA; mingyoungjeng@ku.edu (M.J.); islam.alvir@ku.edu (A.N.); vinayakjha@ku.edu (V.J.); david.levy@ku.edu (D.L.); dckneidel@ku.edu (D.K.); manu.chaudhary@ku.edu (M.C.); ishraq@ku.edu (I.I.); audfacer@ku.edu (A.F.); singhmanish5208@ku.edu (M.S.); evan.baum@ku.edu (E.B.); eade.vanderhoof@ku.edu (E.V.); abina.arshad@ku.edu (A.A.)

**Keywords:** convolutional neural networks, quantum computing, variational quantum algorithms, quantum machine learning

## Abstract

Quantum computing (QC) has opened the door to advancements in machine learning (ML) tasks that are currently implemented in the classical domain. Convolutional neural networks (CNNs) are classical ML architectures that exploit data locality and possess a simpler structure than a fully connected multi-layer perceptrons (MLPs) without compromising the accuracy of classification. However, the concept of preserving data locality is usually overlooked in the existing quantum counterparts of CNNs, particularly for extracting multifeatures in multidimensional data. In this paper, we present an multidimensional quantum convolutional classifier (MQCC) that performs multidimensional and multifeature quantum convolution with average and Euclidean pooling, thus adapting the CNN structure to a variational quantum algorithm (VQA). The experimental work was conducted using multidimensional data to validate the correctness and demonstrate the scalability of the proposed method utilizing both noisy and noise-free quantum simulations. We evaluated the MQCC model with reference to reported work on state-of-the-art quantum simulators from IBM Quantum and Xanadu using a variety of standard ML datasets. The experimental results show the favorable characteristics of our proposed techniques compared with existing work with respect to a number of quantitative metrics, such as the number of training parameters, cross-entropy loss, classification accuracy, circuit depth, and quantum gate count.

## 1. Introduction

The choice of an appropriate machine learning model for specific applications requires consideration of the size of the model since it is linked to the performance [1]. Considering the aforementioned factor, convolutional neural networks (CNNs)s are preferable to multi-layer perceptrons (MLPs)s because of their smaller size and reduced training time while maintaining high accuracy [2,3]. Preserving the spatiotemporal locality of data allows CNNs to reduce unnecessary data connections and therefore reduces their memory requirements [2,3]. This phenomenon reduces the number of required training parameters and thus incurs less training time [2,3].

In the context of quantum computing, great emphasis has been given to quantum-based machine learning, and, in recent years, various techniques have been devised to develop this field [4]. The contemporary quantum machine learning (QML) techniques can be considered as hybrid quantum–classical variational algorithms [5,6,7,8,9]. Generally, variational quantum algorithms (VQAs) utilizes parameterized rotation gates in fixed quantum circuit structures, usually called ansatz, and is optimized using classical techniques like gradient descent [5,6,7,8,9]. However, like MLPs, preserving data locality is challenging for QML algorithms. For instance, the multidimensionality of input datasets is ignored in contemporary QML algorithms and are flattened into one-dimensional arrays [5,6,7,8,9]. Furthermore, the absence of a generalizable technique for quantum convolution limits the capability of QML algorithms to directly adapt CNN structures.

In this work, we present an multidimensional quantum convolutional classifier (MQCC) to address the shortcomings of the existing CNN implementations in reconciling the locality of multidimensional input data. The proposed VQA technique leverages quantum computing to reduce the number of training parameters and time complexity compared with classical CNN models. Similar to the CNN structures, MQCC contains a sequence of convolution and pooling layers for multifeature extraction from multidimensional input data and a fully connected layer for classification.

The subsequent sections of this paper are organized in the following structure. Section 2 discusses fundamental background information regarding different basic and complex quantum operations. Section 3 highlights existing works that are related to the proposed techniques. The proposed methodology is introduced in Section 4 with details given to the constituent parts. The experimental results and the explanation of the used verification metrics are presented in Section 5. Further discussion about the obtained results is provided in Section 6. Finally, Section 7 concludes this work with potential future directions.

## 2. Background

In this section, we present background information pertaining to quantum computing and quantum machine learning. Here, we present the quantum gates and operations that are utilized in the proposed multidimensional quantum convolutional classifier (MQCC). In addition, interested readers may find fundamental details related to quantum information and computing in Appendix A.

### 2.1. Quantum Measurement and Reset

**Figure 1:**
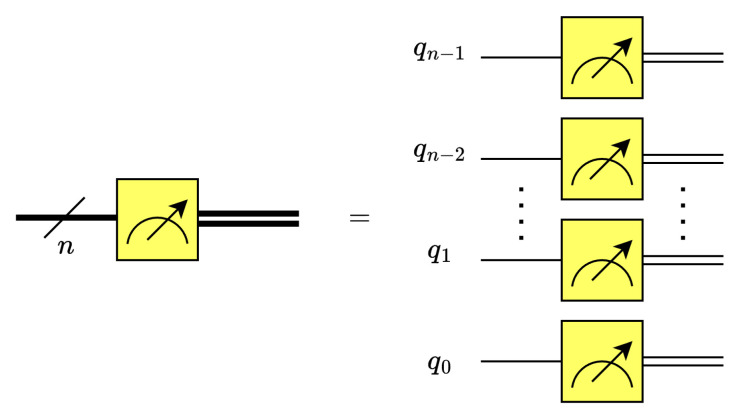
Full quantum state measurement diagram.

The quantum measurement operation of a qubit is usually and informally referred to as a measurement “gate”. The measurement gate is a nonunitary operation that can project the quantum state of a qubit |ψ〉 to the |0〉 or |1〉 basis states [10]. The likelihood of measuring any basis state can be obtained by taking the squared magnitude of their corresponding basis state coefficient. For an *n*-qubit register |ψ〉 with $2^{n}$ possible basis states, the probability of measuring each qubit in any particular basis state |*j*〉, where $0\leq j<2^{n}$ is given by $|c_{j}{|}^{2}$ [11]. The classical output of *n*-qubit amplitude-encoded [12] data can be decoded as $\psi_{\mathrm{decoded}-\mathrm{data}}^{\mathrm{classical}}$. This classical output vector can be reconstructed by the square root of the probability distribution $\sqrt{P(|\psi \rangle )}$, as shown in (1), (2), and Figure 1. When amplitude-encoding [12] is used for encoding positive real classical data, the coefficients of the corresponding quantum pure state [10]|ψ〉 are also positive real, i.e.,  $c_{j}\in R^{+}$, where $0\leq j<2^{n}$. Thus, the amplitudes of |ψ〉 are numerically equal in values to the coefficients of $\psi_{\mathrm{decoded}-\mathrm{data}}^{\mathrm{classical}}$, i.e., $|\psi \rangle =\psi_{\mathrm{decoded}-\mathrm{data}}^{\mathrm{classical}}$. Therefore, the quantum state |ψ〉 can be completely determined from the measurement probability distribution such that $|\psi \rangle =\sqrt{P(|\psi \rangle )}$ only when the amplitudes of the quantum state are all of positive real values. Moreover, the probability distribution P(|ψ〉) can be reconstructed by repeatedly measuring (sampling) the quantum state |ψ〉. In general, an order of $2^{n}$ measurements is required to accurately reconstruct the probability distribution. In order to reduce the effects of quantum statistical noise, it is recommended to gather as many circuit samples (shots) [13] as possible.

(1) $$P\left(|\psi \rangle \right)=[\begin{array}{c} p_{0}\\ p_{1}\\ \vdots \\ p_{j}\\ \vdots \\ p_{N-2}\\ p_{N-1} \end{array}]=[\begin{array}{c} |c_{0}{|}^{2}\\ |c_{1}{|}^{2}\\ \vdots \\ |c_{j}{|}^{2}\\ \vdots \\ |c_{N-2}{|}^{2}\\ |c_{N-1}{|}^{2} \end{array}],\;\mathrm{where}\;p_{j}={\left|c_{j}\right|}^{2},\;\mathrm{and}\;0\leq j<N$$

(2) $$\;\psi_{\mathrm{decoded}-\mathrm{data}}^{\mathrm{classical}}=\sqrt{P\left(|\psi \rangle \right)}=[\begin{array}{c} |c_{0}|\\ |c_{1}|\\ \vdots \\ |c_{j}|\\ \vdots \\ |c_{N-2}|\\ |c_{N-1}| \end{array}]$$

**Figure 2:**
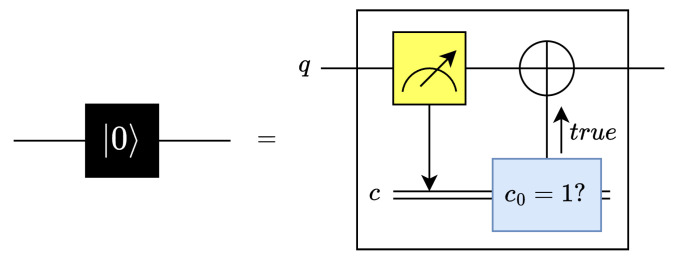
Reset gate and equivalent circuit.

The reset operation sets the state of qubits to |0〉. This operation consists of a midcircuit measurement gate followed by a conditional X gate [14,15] such that the bit-flip operation is applied when the measured qubit is in state |1〉. The reset gate and its equivalent circuit are both shown in Figure 2 [15].

### 2.2. Classical-to-Quantum (C2Q)

**Figure 3:**
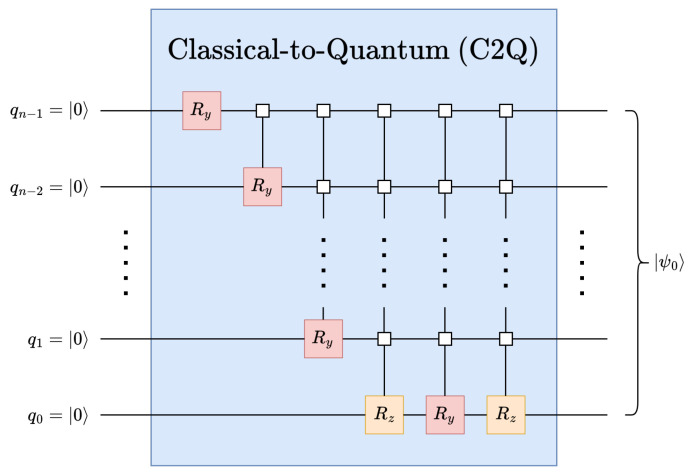
The quantum circuit for classical-to-quantum (C2Q) arbitrary state synthesis [16].

There are a number of quantum data encoding techniques [12,16], each of which uses different methods to initialize quantum circuits from the ground state. Among the many methods, this work leverages the classical-to-quantum (C2Q) arbitrary state synthesis [12,16] operation to perform amplitude encoding and initialize an *n*-qubit state $|\psi_{0}\rangle$, see Figure 3. The C2Q operation employs a pyramidal structure of multiplexed $R_{y}$ and $R_{z}$ gates. It should be noted that the $R_{z}$ gates are only required for positive real data. Thus, for positive real data, the circuit depth is $2\cdot 2^{n}-n-2$, while for complex data, the circuit depth is $3\cdot 2^{n}-n-4$ [12].

### 2.3. Convolutional Neural Networks (CNNs)

CNNs are one of the most widely used types of deep neural networks for image classification [17]. It consists of convolutional, pooling, and fully connected layers. The convolutional layer applies multiple filters to the input to create feature maps. The pooling layer reduces the dimensionality of each feature map but retains the most important information. Some of the most important pooling techniques include max-pooling, average pooling, and sum pooling. Fully connected layers are in the last layers of the CNN network, and their inputs correspond to the flattened one-dimensional matrix generated by the last pooling layer. Activation functions, which are essential for handling complex patterns, are used throughout the network. Finally, a softmax prediction layer is used to generate probability values for each of the possible output labels. The label with the highest probability is selected as the final prediction.

### 2.4. Quantum Machine Learning with Variational Algorithms

Variational quantum algorithms are a type of quantum–classical technique that facilitates the implementations of machine learning on noisy intermediate-scale quantum (NISQ) machines [5,7]. Current quantum devices are not able to maintain coherent states for sufficient periods, preventing current algorithms from performing meaningful optimization on the machine learning model. Thus, VQAs combine classical optimization algorithms with parameterized quantum circuits, or ansatz. Here, the ansatz takes on the role of the model [5]. One specific type of VQA is the variational quantum classifier (VQC), which is used for classification problems. Existing VQCs [6,8,9] have been shown to be effective for classifying datasets with high accuracy and few training parameters in both simulation and current quantum processors.

## 3. Related Work

In this section, we discuss the existing related works with an emphasis on quantum machine learning. Our discussion focuses on commonly used data encoding techniques, existing implementations of the convolution and quantum convolution algorithms, and related quantum machine learning (QML) algorithms. Moreover, we also discuss existing quantum convolutional classification algorithms that leverage data locality.

### 3.1. Data Encoding

For encoding classical image data into the quantum domain, the commonly used methods are Flexible Representation of Quantum Images (FRQI) [18] and Novel Enhanced Quantum Representation (NEQR) [19]. In FRQI, positional and color information is encoded as amplitude encoding and angle encoding, respectively. In NEQR, positions of the pixels are encoded using amplitude encoding but color information is encoded using basis encoding, where *q* represents the number of bits/qubits allocated for color data. For $N=2^{n}$ data points, in terms of circuit width and depth, FRQI incurs n+1 and $O(4^{n})$, respectively, while NEQR incurs n+q and $O(qn2^{n})$, respectively [20]. Although these techniques are employed in the existing quantum convolution techniques, their disadvantages are discussed below.

### 3.2. Convolution

We now discuss existing implementations of convolution and discuss their associated time complexity. These implementations consist of various classical and quantum techniques. In addition, we consider the shortcomings of existing quantum convolution methods.

#### 3.2.1. Classical Convolution

Classical implementations of convolution are usually implemented directly, through general matrix multiplication (GEMM) or through the Fast Fourier transform (FFT). A data size *N*, running the direct implementation on CPUs, has complexity $O(N^{2})$ [21], while the complexity of an FFT-based implementation is O(NlogN) [21]. On GPUs, FFT-based convolution incurs a similar O(NlogN) complexity [22], while the direct approach requires $O(N_{K}N)$ FLOPS [23,24], where $N_{K}$ is the filter size.

#### 3.2.2. Quantum Convolution

The existing quantum convolution techniques [25,26,27,28,29] rely on fixed filter sizes and support only specific filters at a time, e.g., edge detection. They do not contain methods for implementing a general filter. Additionally, these techniques have a quadratic circuit depth, i.e., $O(n^{2})$, where $n=\lceil \log_{2}N\rceil$ is the number of qubits and *N* is the size of the input data. While these methods appear to show quantum advantage, these results do not include overhead incurred from data encoding. The related methods employ the FRQI and NEQR data encoding methods, leading to inferior performance compared with classical methods once the additional overhead is factored in. The authors in [30] propose an edge-detection technique based on quantum wavelet transform QWT and amplitude encoding, named quantum Hadamard edge detection (QHED), which is not generalizable for multiple convolution kernels or multidimensional data. Thus, their algorithm loses parallelism, increases circuit depth, and is difficult to generalize beyond capturing 1-D features. In [20], the authors have developed a quantum convolution algorithm that supports single feature/kernel and multidimensional data. In this work, we leverage the convolution method from [20] and generalize it to support multiple features/kernels in our proposed MQCC framework.

### 3.3. Quantum Machine Learning

There exist two primary techniques for quantum convolutional classification that may leverage data locality through convolution: quantum convolutional neural network (QCNN)s [31] and quanvolutional neural networks [32]. QCNNs are inspired by classical convolutional neural networks, employing quantum circuits to perform convolutions, and quanvolutional neural networks replace classical convolutional layers with quantum convolutional (or quanvolutional) layers.

#### 3.3.1. Quantum Convolutional Neural Networks

The QCNN [31] and our proposed multidimensional quantum convolutional classifier (MQCC) are both VQAs models with structures inspired by CNNs. However, QCNNs borrow the superficial structure of CNNs without considering the underlying purpose. Specifically, the QCNN’s quantum ansatz is designed so that its “convolution” and “pooling” operations exploit the locality of qubits in the circuit (rather than the locality of data). However, unlike data locality, qubit locality does not have a practical purpose for machine learning in terms of isolating relevant input features. Moreover, by considering input data as 1-D, QCNNs do not leverage the dimensionality of datasets, which constitute a primary advantage of CNNs. MQCC, on the other hand, faithfully implements CNN operations in quantum circuits, offering performance improvements in circuit execution time (based on circuit depth) classification accuracy over contemporary implementations of QCNNs on classical computers.

#### 3.3.2. Quanvolutional Neural Networks

Quanvolutional neural networks [32] are a hybrid quantum–classical algorithm named eponymously after the quanvolutional layer added to a conventional CNN. These quanvolutional layers serve to decimate a 2D image, which is then sequentially fed into a quantum device. In this manner, the quanvolutional layer effectively exploits data locality. Yet, the model’s dependency on classical operations, specifically the decimation of input data and the repeated serial data I/O transfer, vastly increases compute time. In contrast, the required convolution operation is incorporated into our proposed MQCC, reducing classical–quantum data transfer. Moreover, MQCC takes advantage of parallelism inherent to quantum computers, while quanvolutional neural networks do not. Together, this allows the MQCC to apply convolutional filters to window data in parallel.

## 4. Materials and Methods

In this section, we describe the materials and methods associated with the proposed multidimensional quantum convolutional classifier (MQCC). The proposed method mainly uses generalized quantum convolution, quantum pooling based on the quantum Haar transform (QHT) and partial measurement [11], and a quantum fully connected layer that is illustrated in this section. To the best of our knowledge, this work is the first to carry out the following:

- Develop a generalizable quantum convolution algorithm for a quantum-convolution-based classifier that supports multiple features/kernels.
- Design a scalable MQCC that uses multidimensional quantum convolution and pooling based on the QHT. This technique reduces training parameters and time complexity compared with other classical and quantum implementations.
- Evaluate the MQCC model in a state-of-the-art QML simulator from Xanadu using a variety of datasets.

### 4.1. Quantum Fully Connected Layer

A fully connected classical neural network constitutes a collection of layers that each perform a linear transformation on $N_{\mathrm{in}}$ input features $x\in R^{N_{\mathrm{in}}}$ to generate $N_{\mathrm{out}}$-feature output $y\in R^{N_{\mathrm{out}}}$ [2,3]. Each layer can be represented in terms of a multiply-and-accumulate (MAC) operation and an addition operation, as shown in (3), where $W\in R^{N_{\mathrm{out}}\times N_{\mathrm{in}}}$ and $b\in R^{N_{\mathrm{out}}}$ represent the trainable weight and bias parameters, respectively. Here, we use bold symbols to represent classical quantities, e.g., vectors, while the Dirac notation to represent their quantum counterparts.

(3) $$y=W^{T}x+b$$

The particular weights and biases that generate the $j^{\mathrm{th}}$ feature of the output, $y_{j}$, can be isolated by taking the $j^{\mathrm{th}}$ column of W, $w_{j}$, and the $j^{\mathrm{th}}$ term of b, $b_{j}$, as shown in (4), which can be directly implemented using quantum circuits. Section 4.1.1 discusses the quantum circuits for a single-feature output, and Section 4.1.2 generalizes the proposed technique for an arbitrary amount of features in the output.

(4) $$\begin{array}{c} y_{j}=\left(w_{j}\cdot x\right)+b_{j},\;\mathrm{where}\;0\leq j<N_{\mathrm{out}} \end{array}$$

#### 4.1.1. Single-Feature Output

For a single-feature output neural network, the weight parameters can be represented as a vector $w\in R^{N_{\mathrm{in}}}$. Here, w can be expressed as a quantum state |*w*〉, as shown in (5), similar to the process of C2Q data encoding; see Section 2.2.

(5) |w〉=1||w||w0⋮0wNin0⋮02nin,wherenin=⌈log2Nin⌉

Similarly, for a single-feature output, Dirac notation of the MAC operation follows from (4), as shown in (6), where |ψ〉 corresponds to the input data.

(6) y−b=〈w|ψ〉

**Figure 4:**
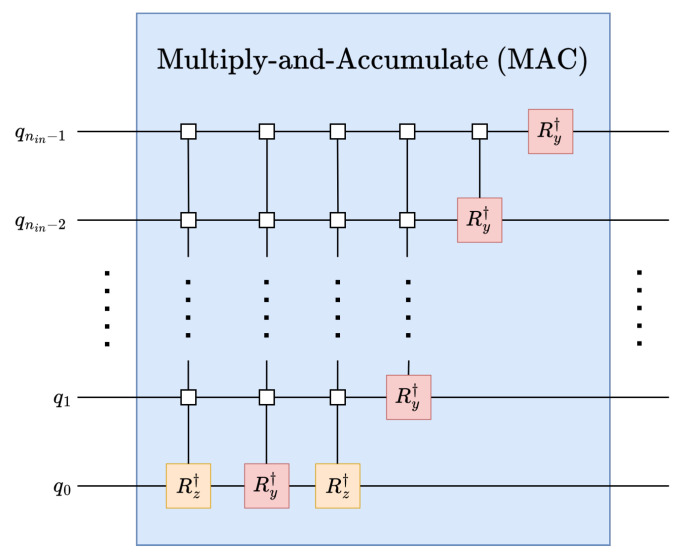
Quantum multiply-and-accumulate (MAC) operation using inverse arbitrary state synthesis.

However, it is necessary to obtain a quantum operator to perform a parameterized unitary linear transformation from the weights vector |*w*〉 on the input data |ψ〉 using an inverse C2Q operation as shown in Figure 4 and described by (7).

(7) $$\begin{array}{cc} U_{\mathrm{MAC}}(|w\rangle ) & =\left[\begin{array}{c} \langle w|\\ \langle \times |\\ \vdots \\ \langle \times | \end{array}\right]↕2^{n_{\mathrm{in}}}\\ \; & =U_{C2Q}^{†}(|w\rangle ) \end{array}$$

#### 4.1.2. Multifeature Output

A multifeature output can be implemented in a naive approach using Single-Feature Output (7) for an $N_{\mathrm{out}}$-feature output, where $N_{\mathrm{out}}\leq N_{\mathrm{in}}$, which can be obtained by encoding each weight vector $w_{j}:0\leq j<N_{\mathrm{out}}$ as a normalized row in $U_{\mathrm{MAC}}$. However, the result might yield a nonunitary operator as the weight vectors can be arbitrary. $U_{\mathrm{MAC}}$ is unitary when each row is orthogonal to all other rows in the matrix, mathematically, $\langle W_{i}|W_{j}\rangle =\delta_{ij}:\forall i,j\in \left[0,N_{\mathrm{out}}\right)$. As described in Appendix A.4, independently defined weights can be supported for each feature of the output by multiplexing $U_{\mathrm{MAC}}$. Now, the generic fully connected operation, $U_{\mathrm{FC}}$, can be generated as shown in (8), where $n_{\mathrm{out}}=\left\lceil \log_{2}N_{\mathrm{out}}\right\rceil$.   

(8) $$U_{\mathrm{FC}}=\left[\begin{array}{ccc} U_{\mathrm{MAC}}(|w_{0}\rangle ) & \; & \\ \; & \ddots & \\ \; & \; & U_{\mathrm{MAC}}(|w_{2^{n_{\mathrm{out}}}-1}\rangle ) \end{array}\right]$$

By generating $N_{\mathrm{out}}$ replicas of the initial state, $|\psi_{0}\rangle$, the operation can be parallelized, i.e., $U_{\mathrm{MAC}}(|w_{0}\rangle )\ldots U_{\mathrm{MAC}}(|w_{2^{n_{\mathrm{out}}}-1}\rangle )$ transformations from (8).

##### Replication:

**Figure 5:**
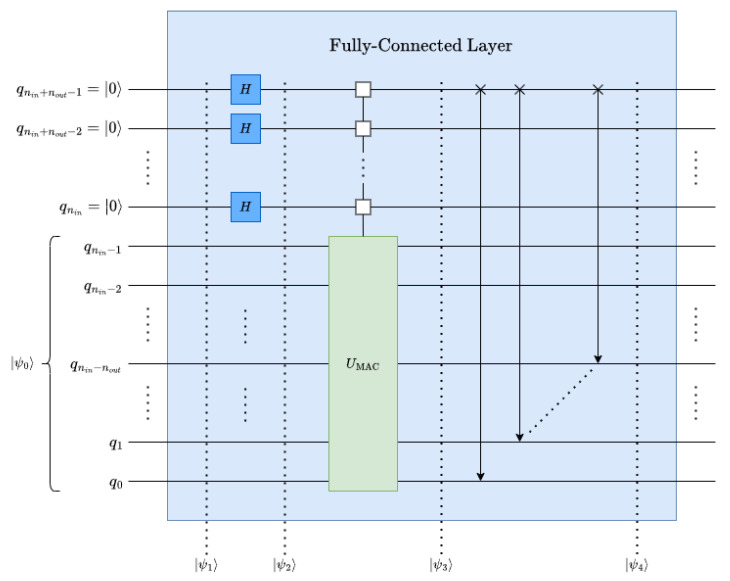
Quantum fully connected layer with an $N_{\mathrm{out}}$-feature output.

To replicate the initial state, $|\psi_{0}\rangle$ $n_{\mathrm{out}}$ qubits, which extends the state vector to a total size of $2^{n_{\mathrm{in}}+n_{\mathrm{out}}}$, see (9) and Figure 5.

(9) |ψ1〉=|0〉⊗nout⊗|ψ0〉=|ψ0〉0⋮0|ψ0〉2nin0⋮02nin+nout

By applying an $n_{\mathrm{out}}$-qubit Hadamard (see Appendix A.3) operation to the relevant qubits the replicas can be obtained through superposition (see (10) and Figure 5), which generates the desired replicas scaled by a factor of $\frac{1}{\sqrt{2^{n_{\mathrm{out}}}}}$ to maintain normalization $\langle \psi_{2}|\psi_{2}\rangle =1$.

(10) |ψ2〉=H⊗nout⊗I⊗nin|ψ1〉=12nout|ψ0〉⋮|ψ0〉|ψ0〉2nin⋮↕⋮|ψ0〉2nin2nin+nout

##### Applying the $U_{\mathrm{FC}}$ Filter:

$U_{\mathrm{FC}}$ can perform the MAC operation for the entire $N_{\mathrm{out}}$-feature output in parallel with the set of replicas of $|\psi_{0}\rangle$; see (11) and Figure 5.

(11) |ψ3〉=UFC|ψ2〉=UMAC(|w0〉)|ψ0〉⋮UMAC(|wj〉)|ψ0〉⋮UMAC(|w2nout−1〉)|ψ0〉UMAC(|w0〉)|ψ0〉2nin⋮↕⋮UMAC(|wj〉)|ψ0〉2nin⋮↕⋮UMAC(|w2nout−1〉)|ψ0〉2nin2nin+nout=〈w0|ψ0〉×⋮×⋮〈wj|ψ0〉×⋮×⋮〈w2nout−1|ψ0〉×⋮×〈w0|ψ0〉×⋮×2nin⋮↕⋮〈wj|ψ0〉×⋮×2nin⋮↕⋮〈w2nout−1|ψ0〉×⋮×2nin2nin+nout

##### Data Rearrangement:

The data rearrangement operation can be performed by applying perfect-shuffle gates; see Appendix A.6. It simplifies the output-feature extraction by gathering them into $N_{\mathrm{out}}$ data points at the top of the state vector; see (12) and Figure 5.

(12) |ψ4〉=∏i=0nout−1RoL(nin+nout−i)⊗I⊗i|ψ3〉=〈w0|ψ0〉⋮〈wj|ψ0〉⋮〈w2nout−1|ψ0〉×⋮×〈w0|ψ0〉⋮〈wj|ψ0〉⋮〈w2nout−1|ψ0〉2nout×⋮×2nin+nout

**Figure 6:**
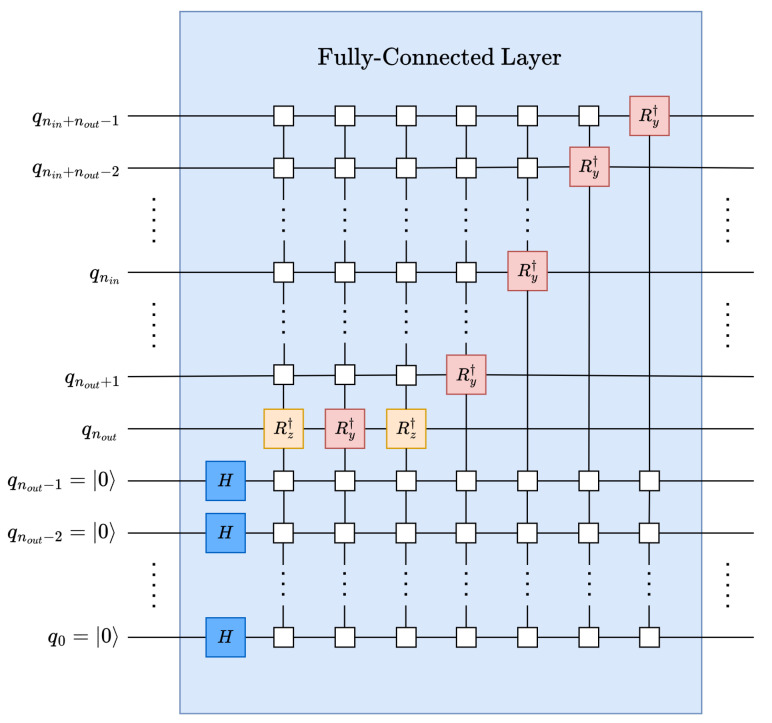
Decomposed and simplified quantum fully connected layer.

It is worth mentioning that instead of applying the auxiliary qubits at the most significant position, as shown in the decomposed and simplified fully connected circuit in Figure 6, auxiliary qubits can be applied at the least significant position to avoid perfect-shuffle permutations.

#### 4.1.3. Circuit Depth of the Quantum Fully Connected Layer

As discussed in Section 2.2, $U_{\mathrm{MAC}}$ operation is implemented by applying the C2Q/arbitrary state synthesis operation with a depth of $\left(3\cdot 2^{n_{\mathrm{in}}}-n_{\mathrm{in}}-4\right)$ fundamental single-qubit and CNOT gates. The depth is expected to increase by a factor of $2^{n_{\mathrm{out}}}$ when multiplexing $U_{\mathrm{MAC}}$ to implement an $N_{\mathrm{out}}$-feature output [33], see (13).

(13) $$\begin{array}{cc} \Delta_{U_{\mathrm{FC}}}\left(n_{\mathrm{in}},n_{\mathrm{out}}\right) & =2^{n_{\mathrm{out}}}\cdot \Delta_{U_{\mathrm{MAC}}\left(n_{\mathrm{in}}\right)}\\ \; & =2^{n_{\mathrm{out}}}\left(3\cdot 2^{n_{\mathrm{in}}}-n_{\mathrm{in}}-4\right)\\ \; & =O(2^{n_{\mathrm{in}}+n_{\mathrm{out}}}) \end{array}$$

### 4.2. Generalized Quantum Convolution

The most significant part of the MQCC framework is the generalized quantum convolution operation with support for arbitrary, parameterized filters. Compared with the classical convolution operation, the convolution operation in the quantum domain achieves exponential improvement in time complexity due to its innate parallelism. The convolution operation consists of stride, data rearrangement, and multiply-and-accumulate (MAC) operations.

#### Stride:

**Figure A8:**
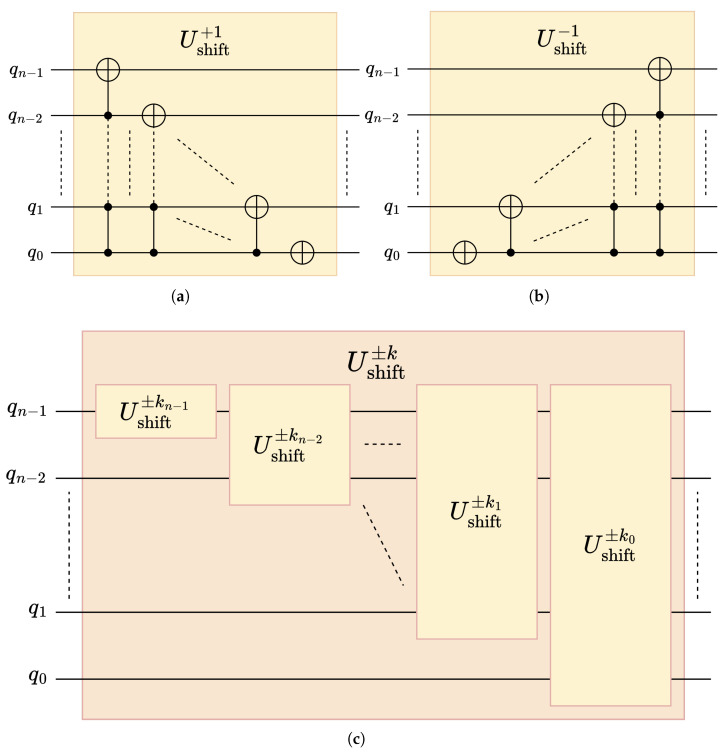
Quantum incrementer, decrementer, and construction of quantum shift operation: (**a**) quantum incrementer by 1; (**b**) quantum decrementer by 1; (**c**) quantum shift operation by ±k, where $k=\sum_{0}^{j=n-1}k_{j}2^{j}$.

The first step of quantum convolution is generating shifted replicas of the input data. Quantum decrementers controlled by additional qubits, called “filter” qubits are used for this purpose. The $U_{\mathrm{shift}}^{-1}$ operator shown in Figure A8b shifts the replica by a single stride.

The stride operation, $U_{\mathrm{stride}}$, is composed of controlled quantum decrementers, where each $U_{\mathrm{shift}}^{-1}$ operation has a quadratic depth complexity; see (A15). Thus, the depth of the controlled quantum decrementer can be derived according to (14), where *n* corresponds to the number of qubits the decrementer is applied to and *c* reflects the number of control qubits.

(14) $$\begin{array}{cc} \Delta_{{\mathrm{cU}}_{\mathrm{shift}}^{\pm 1}}\left(n,c\right)\; & =\sum_{i=c}^{n+c-1}\Delta_{\mathrm{MCX}}\left(i\right)\\ \; & =\sum_{i=c}^{n+c-1}\left(48i-196\right)\\ \; & =\sum_{i=0}^{n-1}48i+48c-196n\\ \; & =24n^{2}+48c-220n\\ \; & =O(n^{2}c)\;\mathrm{for}\;\mathrm{large}\;n \end{array}$$

#### Multiply-and-Accumulate (MAC):

Kernels are applied to the strided replicas of the input data in parallel using the MAC operation; see Figure 4. In the MAC operations, kernels are applied to the contiguous set of data with the help of the inverse arbitrary state synthesis operation. One benefit achieved by using this MAC operation is the superposition of multiple kernels. The superposition of the kernel can be helpful for the classification of multiple features.

#### Data Rearrangement:

Data rearrangement is required to coalesce the output pieces of the MAC steps and create one contiguous piece of output. This step is performed using perfect shuffle permutation (PSP) operations described in Appendix A.6.

#### 4.2.1. One-Dimensional Multifeature Quantum Convolution

The one-dimensional quantum convolution operation, with a kernel of size $N_{K}$ terms, requires generating $N_{K}$ replicas of the input data in a range of possible strides between $0\leq k<N_{K}$. Therefore, a total of $N_{K}N$ terms need to be encoded into a quantum circuit, including the $n_{k}=\left\lceil \log_{2}N_{K}\right\rceil$ additional auxiliary qubits, denoted as “kernel” qubits, which are allocated the most significant qubits to maintain data contiguity.

**Figure 7:**
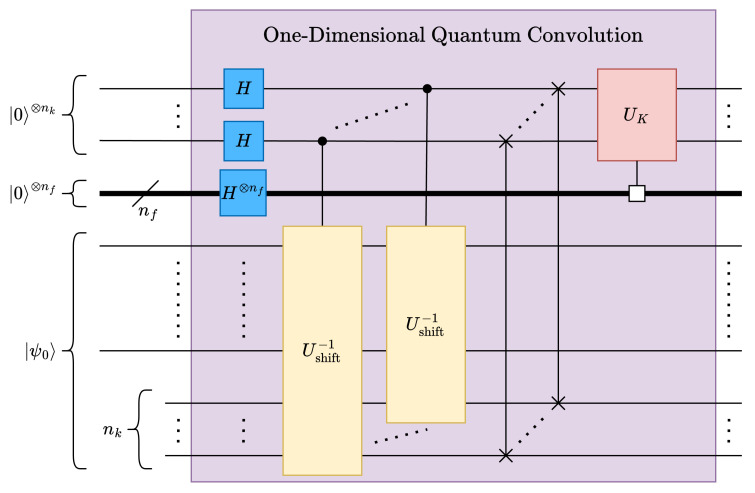
One-dimensional quantum convolution circuit.

Necessary $N_{K}$ replicas of the input vector are created by using Hadamard gates; see Figure 7. Convolution kernels can be implemented using multiply-and-accumulate (MAC) operations; as such, it is possible to leverage $U_{\mathrm{MAC}}$, as defined in Section 4.1, for implementing quantum convolution kernels. Given a kernel $K\in R^{N_{K}}$, the corresponding kernel operation $U_{K}$ can be constructed from the normalized kernel |K〉, as shown in (15).

(15) $$U_{K}=U_{\mathrm{MAC}}(|K\rangle ),\;\mathrm{where}\;|K\rangle =\hat{K}=\frac{K}{\left||K|\right|}$$

When applied to the $n_{k}$ lower qubits of the state vector, $U_{K}$ applies the kernel *K* to all data windows in parallel. However, in CNNs, convolution layers typically must support multiple convolution kernels/features. Fortunately, one major advantage of the proposed quantum convolution technique is that multiple features can be supported by multiplexing only the MAC operations—the stride and data rearrangement operations do not need to be multiplexed; see Figure 7. Accordingly, for $N_{F}$ features, $n_{f}=\left\lceil \log_{2}N_{F}\right\rceil$ must be added to the circuit and placed in superposition using Hadamard gates, similar to the process in (9). Thus, the depth complexity of $U_{\mathrm{stride}}$ can be expressed in terms of $\Delta_{{\mathrm{cU}}_{\mathrm{shift}}^{-1}}\left(n-j,c\right)$, as described by (16), where c=1 for all $0\leq j<n_{k}$; see Figure 7. Similarly, the depth complexity of $U_{K}$ can be expressed by (17). Finally, The depth of the proposed multifeature 1D quantum convolution can be obtained as (18).   

(16) $$\begin{array}{cc} \Delta_{U_{\mathrm{stride}}}\left(n,n_{k}\right) & =\sum_{j=0}^{n_{k}-1}\Delta_{U_{\mathrm{shift}}^{-1}}\left(n-j,1\right)=\sum_{j=0}^{n_{k}-1}\left[24{\left(n-j\right)}^{2}+48-220\left(n-j\right)\right]\\ \; & \leq 24n_{k}n^{2}-24n_{k}^{2}n-196n_{k}n+8n_{k}^{3}-98n_{k}^{2}-58n_{k}\\ \; & =O\left(n_{k}n^{2}-n_{k}^{2}n+n_{k}^{3}\right)\;\mathrm{for}\;\mathrm{large}\;n\gg n_{k} \end{array}$$

(17) $$\begin{array}{cc} \Delta_{U_{K}}\left(n_{k},n_{f}\right) & =2^{n_{f}}\cdot \Delta_{U_{\mathrm{mac}}\left(n_{k}\right)}\\ \; & \leq 2^{n_{f}}\left(3\cdot 2^{n_{k}}-n_{k}-4\right)\\ \; & =O\left(2^{n_{f}+n_{k}}\right)\;\mathrm{for}\;\mathrm{large}\;n_{f},n_{k} \end{array}$$

(18) $$\begin{array}{cc} \Delta_{1D\;\mathrm{conv}}\left(n,n_{k},n_{f}\right)\; & =\Delta_{H}+\Delta_{U_{\mathrm{stride}}}\left(n,n_{k}\right)+\Delta_{U_{K}}\left(n_{k},n_{f}\right)+\Delta_{\mathrm{SWAP}}\\ \; & =1+24n_{k}n^{2}-24n_{k}^{2}n-196n_{k}n+8n_{k}^{3}-98n_{k}^{2}-58n_{k}\\ \; & +2^{n_{f}}\left(3\cdot 2^{n_{k}}-n_{k}-4\right)+3\\ \; & =O\left(n_{k}n^{2}-n_{k}^{2}n+2^{n_{f}+n_{k}}\right),\;\mathrm{where}\;n\gg n_{f},n_{k} \end{array}$$

#### 4.2.2. Multidimensional Multifeature Quantum Convolution

**Figure 8:**
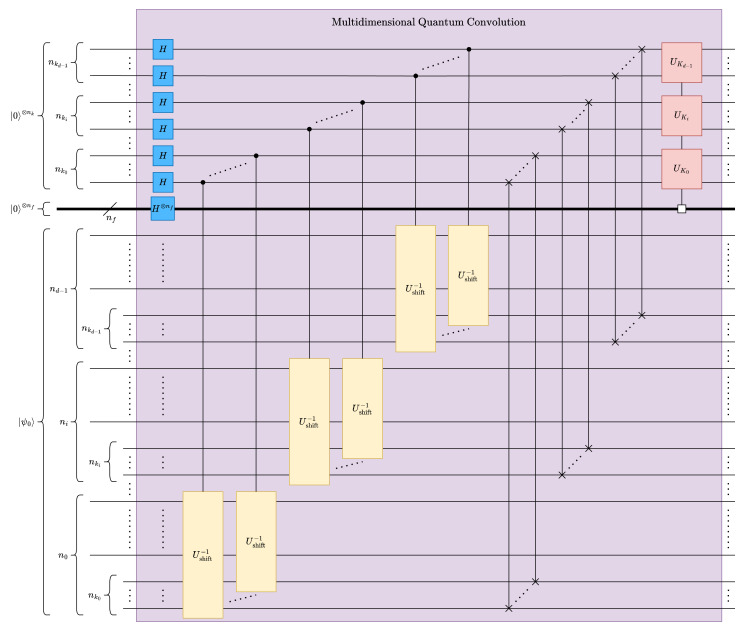
Multidimensional quantum convolution circuit.

Multidimensional quantum convolution can be implemented by stacking multiple one-dimensional quantum circuits, as shown in Figure 8. A *d*-dimensional quantum convolution circuit can be constructed with a stacked kernel of 1-dimensional convolution circuits only when the multidimensional kernels are outer products of *d* instances of 1-dimensional kernels. The depth of *d*-D quantum convolution can be obtained as (19).

(19) $$\begin{array}{cc} \Delta_{d-D\;\mathrm{conv}}\left(n,n_{k},n_{f}\right) & =O\left(n_{k_{\max}}n_{\max}^{2}-n_{k_{\max}}^{2}n_{\max}+2^{n_{k}+n_{f}}\right),\;\mathrm{where}\;\\ n_{\max} & =\max_{i=0}^{d-1}\left(n_{i}\right),\;n_{k_{\max}}=\max_{i=0}^{d-1}\left(n_{k_{i}}\right),\;\mathrm{and}\;n_{\max}\gg n_{f},n_{k_{\max}} \end{array}$$

### 4.3. Quantum Pooling

A critical part of CNNs is the pooling operation or downsampling of the feature maps. One widely used method is average pooling, where only the average of the adjacent pixels in the feature map is preserved, creating a smoothing effect [34].

#### 4.3.1. Quantum Average Pooling using Quantum Haar Transform

The average pooling operation can be implemented using the quantum wavelet transform (QWT) [11], which has the advantage of preserving data locality using wavelet transform decomposition. It is a commonly used technique for dimension reduction in image processing [11]. In this work, we utilize the simplest and first wavelet transform, quantum Haar transform (QHT) [11] to implement quantum pooling operation. This operation is executed in two steps: the Haar wavelet operation and data rearrangement.

##### Haar Wavelet Operation:

For separating the high- and low-frequency components from the input data, H gates are applied in parallel. The number of H gates applied in QHT is equal to the levels of decomposition.

##### Data Rearrangement:

After separating the high- and low-frequency components, quantum rotate-right (RoR) operations are applied to group them accordingly. As mentioned before, the proposed framework is highly parallelizable regardless of the dimensions of the data, as the QHT operation can be applied to multiple dimensions of data in parallel.

**Figure 9:**
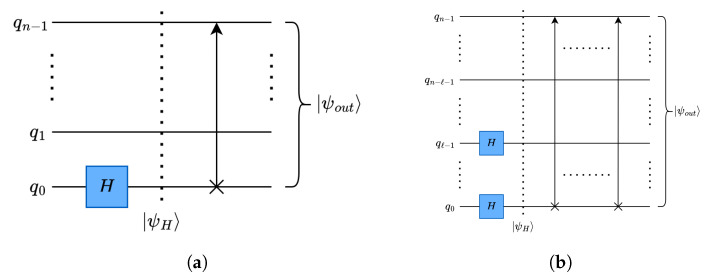
Quantum Haar transform circuits: (**a**) single-level One-dimensional (1-D) QHT circuit; (**b**) multilevel One-dimensional (1-D) QHT circuit.

As shown in Figure 9a, for a single-level of decomposition, H gates are applied on one qubit (the least significant qubit) per dimension, and for *ℓ*-level decomposition, shown in Figure 9b, *l* number of H gates are applied per dimension. Each level of decomposition reduces the size of the corresponding dimension by a factor of 2.

#### 4.3.2. Quantum Euclidean Pooling using Partial Measurement

In machine learning applications, the average and maximum (max) pooling [34] are the most commonly used pooling schemes for dimension reduction. The two schemes differ in the sharpness of data features. On one hand, max pooling yields a sharper definition of input features, which makes it preferable for edge detection and certain classification applications [34]. On the other hand, average pooling offers a smoother dimension reduction that may be preferred in other workloads [34]. Thus, to accompany our implementation of quantum averaging pooling using QHT (see Section 4.3.1), it would be beneficial to have an implementation of quantum max pooling. However, such an operation would be nonunitary, creating difficulty for the implementation of quantum max pooling [35]. Therefore, instead of max pooling, we utilize an alternative pooling technique we denote as quantum Euclidean pooling.

Mathematically, average and Euclidean pooling are special cases of the *p*-norm [36], where for a vector of size *N* elements, the *p*-norm or $\ell^{p}$ norm of a vector $x\in C^{N}$ is given by (20) for p∈Z [36]. The average pooling occurs for the 1-norm (p=1) and Euclidean pooling occurs for the 2-norm (p=2). A notable benefit of the Euclidean pooling technique is its zero-depth circuit implementation by leveraging partial measurement [35].

(20) $${\left||x|\right|}_{p}={\left(\sum_{i=0}^{N}x_{i}^{p}\right)}^{\frac{1}{p}}$$

**Figure 10:**
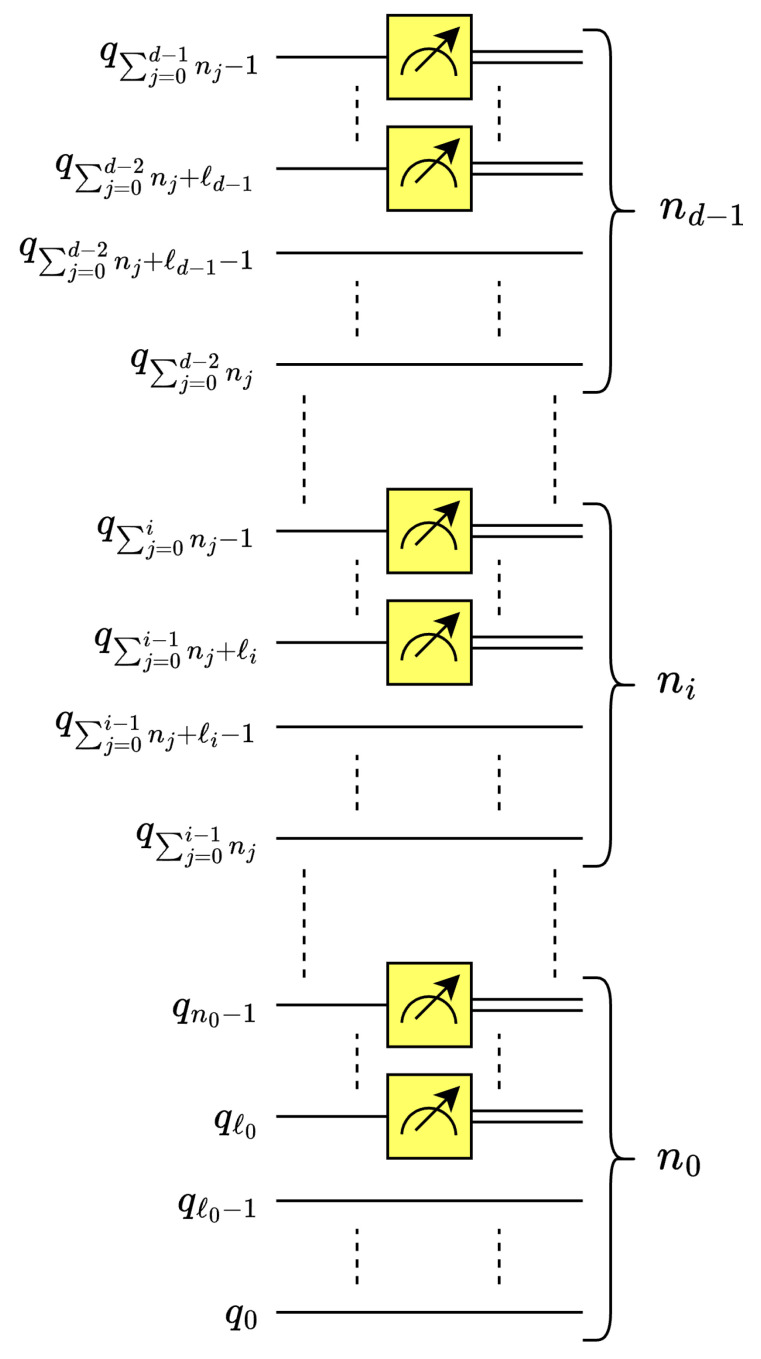
Multilevel, *d*-D Euclidean pooling circuit.

This work leverages the multilevel, *d*-dimensional quantum Euclidean pooling circuit presented in [35]; see Figure 10. Here, for each dimension *i*, $\ell_{i}$ is the number of decomposition levels for dimension where 0≤i<d [35].

### 4.4. Multidimensional Quantum Convolutional Classifier

**Figure 11:**
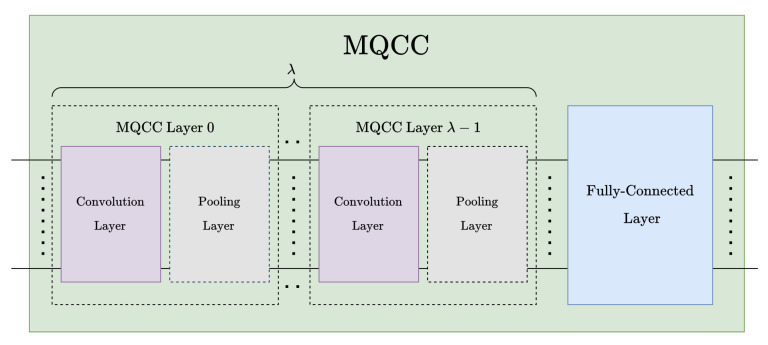
High-level overview of the MQCC architecture.

The proposed multidimensional quantum convolution classifier framework, see Figure 11, resembles a CNN [2] structures. After a sequence of convolution pooling (CP) pairs, the model is finally connected to a fully connected layer, see Figure 6 and Figure 11. The total number of layers in the proposed model can be expressed in terms of CP pairs as 2λ+1, where λ is the number of CP pairs.

It is worth mentioning that there is no advantage in changing the number of features among convolution/pooling layers in the MQCC because of the implementation constraints. Therefore, the total number of kernel features can be estimated globally instead of layer-by-layer.

The circuit width of the MQCC (21) can be derived from the number of convolution layers, pooling layers, and the fully connected layer. Input data are encoded using *n* qubits, and each convolution operation adds $n_{f}=\left\lceil \log_{2}N_{F}\right\rceil$ qubits for $N_{F}$ features and $n_{k}=\left\lceil \log_{2}N_{K}\right\rceil$ qubits per layer for kernels. In addition, the fully connected operation contributes $n_{c}=\left\lceil \log_{2}N_{C}\right\rceil$ qubits to encode $N_{C}$ output features/classes. On the other hand, each Euclidean pooling layer frees *ℓ* qubits, which can then be reused by other layers.

(21) $$\begin{array}{cc} n_{\mathrm{MQCC}}^{\mathrm{Avg}} & =n+n_{f}+\lambda n_{k}+n_{c}\\ n_{\mathrm{MQCC}}^{\mathrm{Euclidean}} & =n+n_{f}+n_{k}+\max\left(0,\left(\lambda -1\right)n_{k}-\lambda \ell +n_{c}\right) \end{array}$$

The MQCC can be further parallelized in terms of circuit depth between the interlayer convolution/fully connected layers. This parallelism can be achieved by performing (multiplexed) MAC operations from the quantum convolution and fully connected layers in parallel with the stride operation from the previous layer(s) of quantum convolution. The circuit depth of MQCC can be derived as shown in (22).

(22) $$\begin{array}{cc} \Delta_{\mathrm{MQCC}} & =\max_{i=0}^{d-1}\Delta U_{\mathrm{stride}}\left(n_{i},n_{k_{i}}\right)+\lambda \left(\Delta_{H}+\Delta_{\mathrm{SWAP}}\right)\\ \; & +\sum_{j=0}^{\lambda -2}\max\left(\max_{i=0}^{d-1}\Delta_{U_{\mathrm{stride}}}\left(n_{i}-j\ell_{i},n_{k_{i}}\right),\Delta_{U_{K}}\left(n_{k},n_{f}\right)\right)\\ \; & +\max\left(\Delta_{U_{\mathrm{FC}}}\left(n-\left(\lambda -1\right)\ell ,n_{c}\right),\Delta_{U_{K}}\left(n_{k},n_{f}\right)\right) \end{array}$$

### 4.5. Optimized MQCC

**Figure 12:**
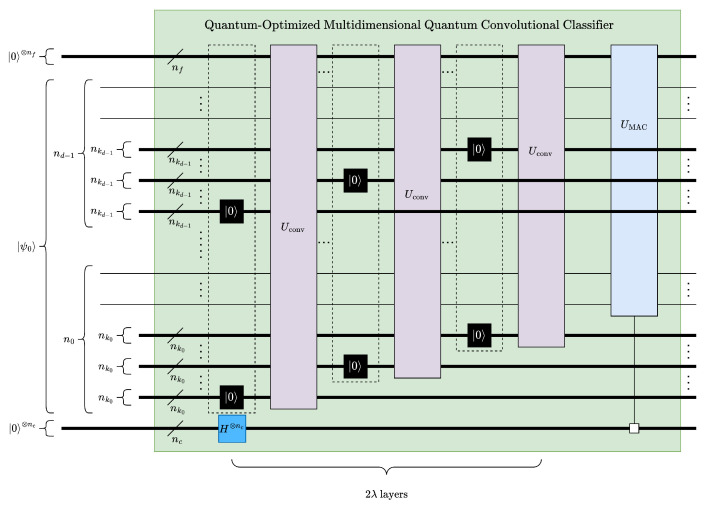
Quantum-Optimized Multidimensional Quantum Convolutional Classifier (MQCC Optimized).

Figure 12 presents a width-optimized implementation of MQCC, which we refer to as Quantum-Optimized Multidimensional Quantum Convolutional Classifier (MQCC Optimized). To reduce the required number of qubits, the convolution and pooling operations are swapped, which allows kernel qubits to be trimmed for each convolution layer, see Section 4.2. To achieve higher processing efficiency, trimmed qubits are reassigned to later layers of dimension reduction and run in parallel. Furthermore, only Euclidean pooling with partial measurements is used because of the inherent circuit depth efficiency. The circuit width of MQCC Optimized is shown in (23), where *n* is the number of qubits corresponding to the data size, $n_{f}$ is the number of qubits corresponding to the features, and $n_{c}$ is the number of qubits corresponding to the classes. If necessary, additional pooling operations can be applied to keep the circuit width at or below the absolute minimum number of qubits *n* by excluding qubits dedicated to features and classes. It should be noted that reordering convolution and pooling operations reduces the maximum number of convolution operations by 1.

(23) $$n_{\mathrm{MQCC}\;\mathrm{Optimized}}=n+n_{f}+n_{c}$$

Accordingly, the depth of MQCC Optimized can be expressed as shown in (24).

(24) $$\begin{array}{cc} \Delta_{\mathrm{MQCC}}^{\mathrm{opt}} & =\max_{i=0}^{d-1}\Delta U_{\mathrm{stride}}\left(n_{i}-\ell_{i},n_{k_{i}}\right)+\lambda \left(\Delta_{H}+\Delta_{\mathrm{SWAP}}\right)\\ \; & +\sum_{j=1}^{\lambda -1}\max\left(\max_{i=0}^{d-1}\Delta_{U_{\mathrm{stride}}}\left(n_{i}-j\ell_{i},n_{k_{i}}\right),\Delta_{U_{K}}\left(n_{k},n_{f}\right)\right)\\ \; & +\max\left(\Delta_{U_{\mathrm{FC}}}\left(n-\lambda \ell ,n_{c}\right),\Delta_{U_{K}}\left(n_{k},n_{f}\right)\right) \end{array}$$

**Figure 13:**
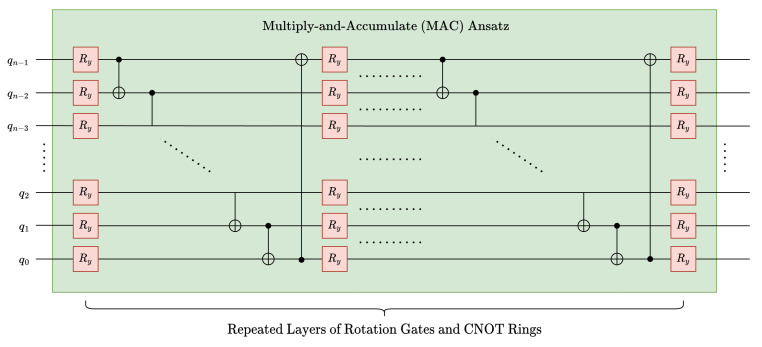
Alternate ansatz $\left(U_{\mathrm{ansatz}}\right)$ option, distinct from $U_{\mathrm{MAC}}$.

To further reduce the depth of MQCC Optimized, we investigated replacing inverse-C2Q operations for MAC operations with different parameterized ansatz. More specifically, a common ansatz in QML, namely NLocal operation in Qiskit [37] or BasicEntanglerLayer in Pennylane [38], was utilized; see Figure 13. The depth of this ansatz is linear with respect to the data qubits (see (25)), which is a significant improvement over arbitrary state synthesis, which has a circuit depth of $O(2^{n})$ for an *n*-qubit state. Although the ansatz could potentially reduce circuit depth, its structure lacks theoretical motivation or guarantees for high fidelity when modeling convolution kernels.

(25) $$\Delta_{\alpha }\left(n,\ell \right)=\ell \cdot n+1$$

## 5. Experimental Work

In this section, we first detail our experimental setup, followed by the results for the proposed MQCC technique. Experiments were conducted using real-world, multidimensional image data to test both the individual and composite components of our techniques.

### 5.1. Experimental Setup

The MQCC methodology was validated by first evaluating its most important component, namely convolution using the metric of fidelity and then evaluating the complete technique, i.e., MQCC and MQCC optimized by conducting classifications experiments using 1D, 2D, and 3D datasets.

For the convolution experiments on 1D data, we used audio material published by the European Broadcasting Union for sound quality assessment [39]. Using a preprocessing step, the data were converted into a single channel, with the data size varying from $2^{8}$ data points to $2^{20}$ data points, sampled at a rate of 44.1 kHz.

**Figure 14:**
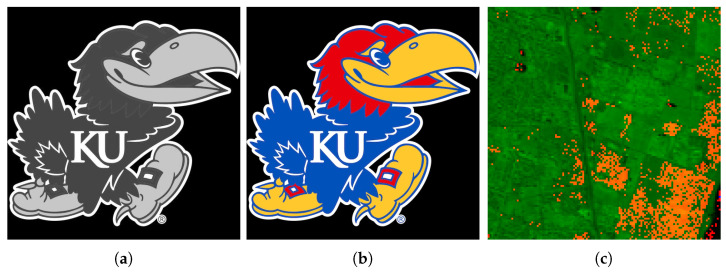
High-resolution, multidimensional, real-world input data used in experimental trials: (**a**) 2D B/W image [40]; (**b**) 3D RGB image [40]; (**c**) 3D hyperspectral image [41].

For conducting 2D convolution experiments, we used 2D images that are either black and white or color Jayhawks [40], as shown in Figure 14. These images range in size from (8×8) pixels to (512×512×3) pixels. For the 3D image experiments, we used hyperspectral images from the Kennedy Space Center (KSC) dataset [41]. The images were preprocessed and resized, with the sizes ranging from (8×8×8) pixels to (128×128×128) pixels. Simulations of quantum convolution operation were run using Qiskit SDK (v0.45.0) from IBM Quantum [13] over the given data. To demonstrate the effect of statistical noise on the fidelity (26), both noise-free and noisy (with 1,000,000 circuit samples/shots) simulation environments were evaluated.

(26) $$\mathrm{Fidelity}\left(X,Y\right)=\frac{\langle X,Y\rangle }{{\left||X|\right|}_{F}{\left||Y|\right|}_{F}}$$

To evaluate the performance of the complete MQCC and the MQCC-optimized technique, they were tested against CNNs, QCNNs, and quanvolutional neural networks by their capabilities of binary classification on real-world datasets, such as MNIST [42], FashionMNIST [43], and CIFAR10 [44]. The classical components in these trials were run using PyTorch (v2.1.0) [45], while the quantum circuits used Pennylane (v0.32.0), a Xanadu QML-focused framework [46].

The experiments were performed on a cluster node at the University of Kansas [47]. The node consisted of a 48-Core Intel Xeon Gold 6342 CPU, three NVIDIA A100 80 GB GPUs (CUDA version 11.7) with PCIe 4.0 connectivity and 256 GB of 3200 MHz DDR4 RAM. To account for initial parameter variance in ML or noise in noisy simulations, experiments were repeated for 10 trials, with the median being displayed in graphs.

### 5.2. Configuration of ML Models

The different techniques fundamentally being ML models meant that they could share some parameters and metrics during their testing. For example, the log loss and the Adam optimizer [48] were shared by all the techniques, and the “feature-count” metric was shared between the CNN and MQCC, which have 4 features per convolution layer. The parameters that were unique to each model are discussed next.

**Figure 15:**
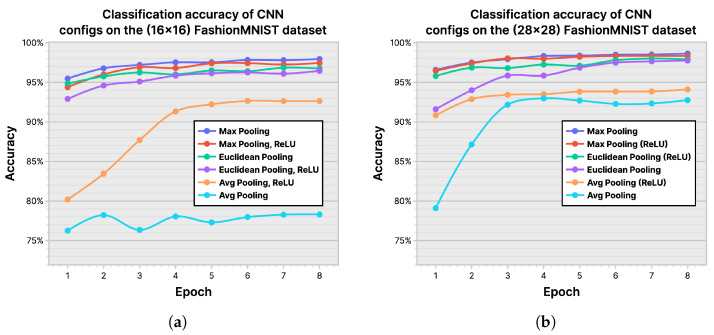
Classification accuracy of convolutional neural network (CNN) configurations on the FashionMNIST dataset: (**a**) (16×16) dataset; (**b**) (28×28) dataset.

***Convolutional Neural Networks:*** In Figure 15, we show the classification accuracy of the CNN model on (16×16) and (28×28) FashionMNIST datasets, using average pooling, max pooling, and Euclidean pooling. The plots show the obtained accuracy with and without ReLU [49], which is an optional layer that can be appended to each pooling layer in a CNN. Based on the results, which show Max Pooling without ReLU to be the configuration with the best accuracy, we chose it to be the baseline configuration for CNN in our tests.

**Figure 16:**
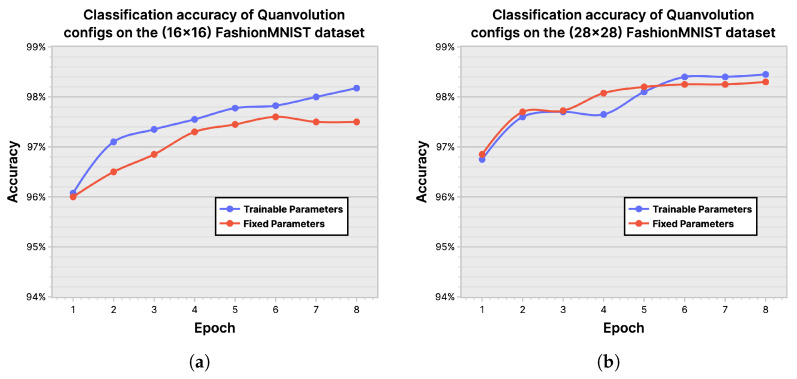
Classification accuracy of quanvolution neural network configurations on the FashionMNIST dataset: (**a**) (16×16) dataset; (**b**) (28×28) dataset.

***Quanvolutional Neural Networks:*** While quanvolutional neural networks were initially introduced without a trainable random quantum circuit in the quanvolutional layer, later work has suggested implementing parameterized and trainable quanvolutional layers. We, therefore, test both the trainable and nontrainable quanvolutional techniques, and Figure 16 demonstrates that the trainable variant outperforms the other method in the (16×16) FashionMNIST dataset, although the differences are negligible on the (28×28) dataset. This is used as evidence behind our decision to use the trainable variant of the quanvolutional neural network as the baseline for comparison with the other models.

**Figure 17:**
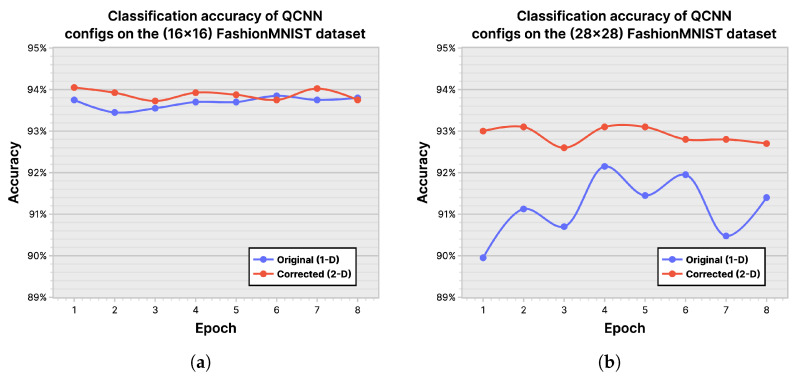
Classification accuracy of quantum convolutional neural network (QCNN) configurations on the FashionMNIST dataset: (**a**) (16×16) dataset; (**b**) (28×28) dataset.

***Quantum Convolutional Neural Networks:*** We based our implementation of the QCNN on [50]; however, some modifications were made to the technique to work around limitations present in the data encoding method. When encoding data that are not of size $2^{n}$ in each dimension, the original method flattens (vectorizes) the input before padding with zeros, as opposed to padding each dimension. However, this sacrifices the advantage of multidimensional encoding, where each dimension is mapped to a region of qubits. To ensure a level field between QCNN and MQCC, the (16×16) and (28×28) FashionMNIST datasets were tested both for the original (1D) and a corrected (2D) data encoding configuration of the QCNN, the results of which are shown in Figure 17. As expected, we see a clear improvement on the (28×28) dataset, and based on this, we chose the corrected (2D) data encoding method as our baseline QCNN for comparison against other ML models.

### 5.3. Results and Analysis

We first present the results of the quantum convolution operations on data with varying dimensionalities. Then, we compare the fidelity results of the quantum convolution under a noisy simulation environment with reference to classical convolution implementation. Finally, we present the results for MQCC.

#### Quantum Convolution Results

The fidelity of the quantum convolution technique was tested in both a noise-free and noisy environment against a classical implementation using common (3×3) and (5×5) filter kernels. These kernels, as described in (27)–(33), include the Averaging $F_{\mathrm{avg}}$, Gaussian blur $F_{\mathrm{blur}}$, Sobel edge-detection $F_{\mathrm{Sx}}$/$F_{\mathrm{Sy}}$, and Laplacian of Gaussian blur (Laplacian) $F_{L}$ filters. To enable a quantum implementation of these kernels, a classical preprocessing step zero-padded each kernel until the dimensions were an integer power of two. As negative values may occur in classical convolution, the magnitudes of the output values were cast into a single-byte range [0,255] in a classical postprocessing step.

(27) $$F_{\mathrm{avg}}^{3\times 3}=\frac{1}{9}\left[\begin{array}{ccc} 1 & 1 & 1\\ 1 & 1 & 1\\ 1 & 1 & 1 \end{array}\right],$$

(28) $$F_{\mathrm{avg}}^{5\times 5}=\frac{1}{25}\left[\begin{array}{ccccc} 1 & 1 & 1 & 1 & 1\\ 1 & 1 & 1 & 1 & 1\\ 1 & 1 & 1 & 1 & 1\\ 1 & 1 & 1 & 1 & 1\\ 1 & 1 & 1 & 1 & 1 \end{array}\right]$$

(29) $$F_{\mathrm{blur}}^{3\times 3}=\frac{1}{16}\left[\begin{array}{ccc} 1 & 2 & 1\\ 2 & 4 & 2\\ 1 & 2 & 1 \end{array}\right],$$

(30) $$F_{\mathrm{blur}}^{5\times 5}=\frac{1}{273}\left[\begin{array}{ccccc} 1 & 4 & 7 & 4 & 1\\ 4 & 16 & 26 & 16 & 4\\ 7 & 26 & 41 & 26 & 7\\ 4 & 16 & 26 & 16 & 4\\ 1 & 4 & 7 & 4 & 1 \end{array}\right]$$

(31) $$F_{\mathrm{Sx}}=\frac{1}{4}\left[\begin{array}{ccc} 1 & 0 & -1\\ 2 & 0 & -2\\ 1 & 0 & -1 \end{array}\right],$$

(32) $$F_{\mathrm{Sy}}=\frac{1}{4}\left[\begin{array}{ccc} 1 & 2 & 1\\ 0 & 0 & 0\\ -1 & -2 & -1 \end{array}\right]$$

(33) $$F_{L}^{3\times 3}=\frac{1}{6}\left[\begin{array}{ccc} 1 & 1 & 1\\ 1 & -8 & 1\\ 1 & 1 & 1 \end{array}\right],$$

(34) $$F_{L}^{5\times 5}=\frac{1}{20}\left[\begin{array}{ccccc} 1 & 1 & 1 & 1 & 1\\ 1 & 1 & 1 & 1 & 1\\ 1 & 1 & -24 & 1 & 1\\ 1 & 1 & 1 & 1 & 1\\ 1 & 1 & 1 & 1 & 1 \end{array}\right]$$

**Figure 18:**
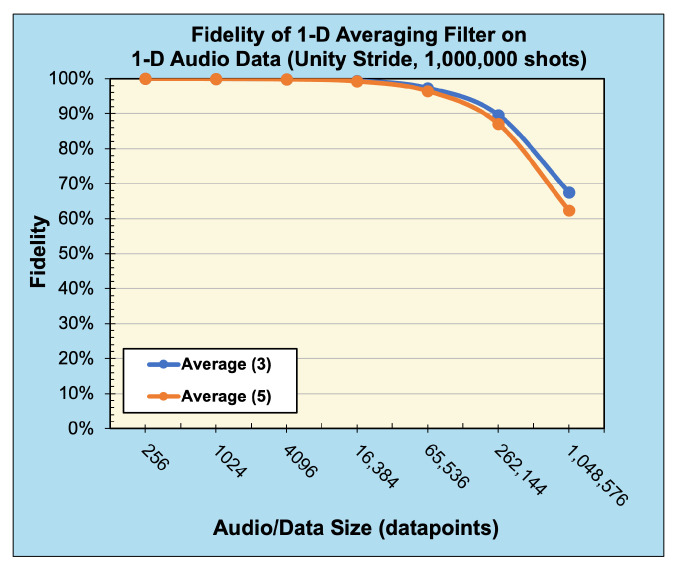
Fidelity of 1D convolution (averaging) filters with unity stride on 1D audio data (sampled with 1,000,000 shots).

A 1D averaging kernel of sizes (1×3) and (1×5) was applied to audio files after preprocessing, described in Section 5.1, with data size ranging from $2^{8}$ to $2^{20}$ data points. Table 1 presents the reconstructed output data of this operation, with Figure 18 displaying the associated calculated fidelity.

The 2D averaging, Gaussian blur, Sobel edge-detection, and Laplacian kernels were applied to 2D black-and-white (B/W) and 3D RGB Jayhawk images, see Figure 14, ranging from (8×8) to (512×512) pixels and (8×8×3) pixels to (512×512×3) pixels, respectively.

The reconstruction from convolution operations in classical, noise-free, and noisy environments on (128×128)- and (128×128×3)-pixel input images can be seen in Table 2 and Table 3, respectively.

Finally, a 3D averaging kernel of sizes (3×3×3) and (5×5×5) was applied to hyperspectral images from the KSC dataset [41]. The images were preprocessed and resized to a power of two, ranging from (8×8×8) pixels to (128×128×128) pixels in size. Table 4 shows the reconstructed output images from convolution operations in classical, noise-free, and noisy environments.

**Figure 19:**
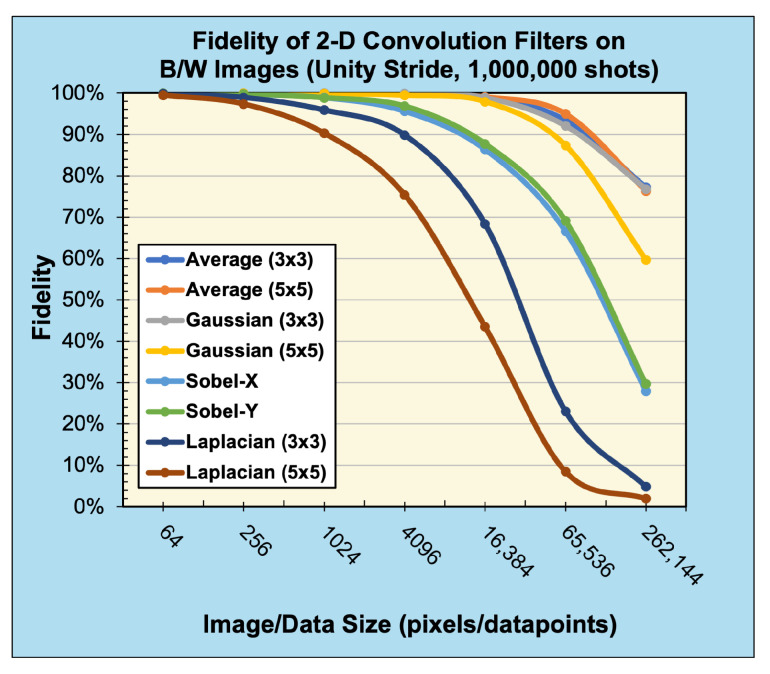
Fidelity of 2D convolution filters with unity stride on 2D B/W data (sampled with 1,000,000 shots).

**Figure 20:**
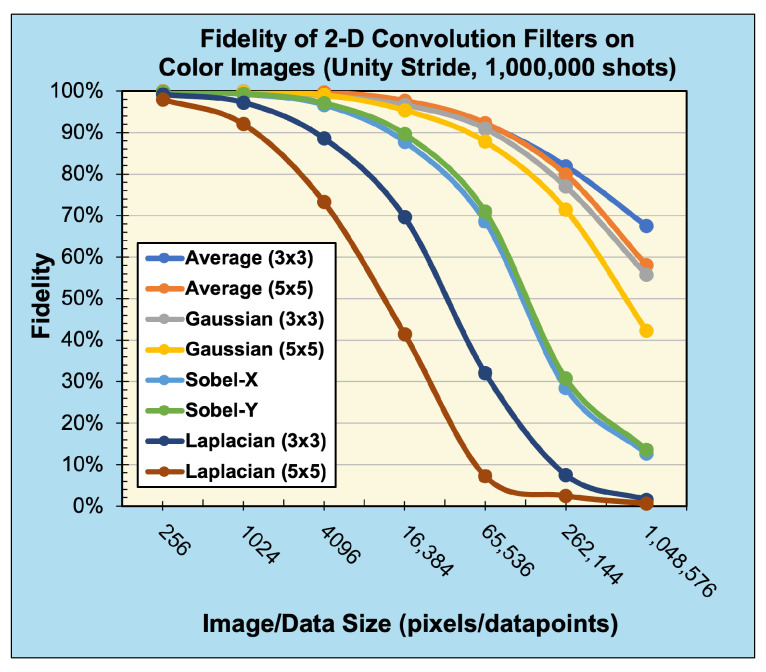
Fidelity of 2D convolution filters with unity stride on 3D RGB data (sampled with 1,000,000 shots).

**Figure 21:**
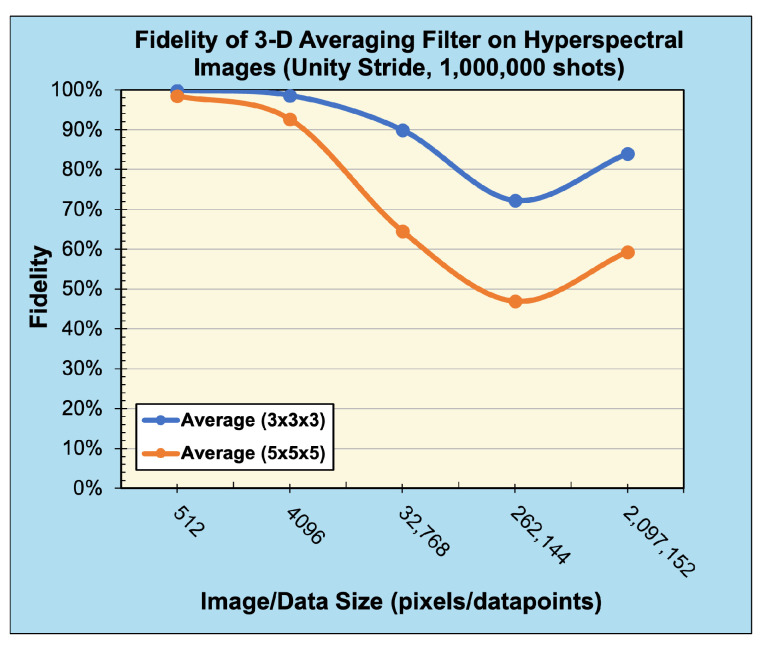
Fidelity of 3D convolution (averaging) filters with unity stride on 3D hyperspectral data (sampled with 1,000,000 shots).

Compared with the expected, classically generated results, the noise-free quantum results tested at 100% fidelity across all trials. Therefore, in a noise-free environment, given the same inputs, the proposed convolution techniques have no degradation compared with classical convolution. The fidelity of noisy simulations using 1D audio, 2D B/W, 2D RGB, and 3D hyperspectral data are presented in Figure 18, Figure 19, Figure 20, and Figure 21, respectively. The fidelity degradation in these figures is due to the statistical noise where the constant shot count (number of circuit samples) becomes less and less sufficient to describe the increasing data size. We could improve this reduction from noise by increasing the number of shots, but our experiments were limited to 1,000,000 shots in simulation, which is the maximum number of shots allowed by the simulator.

## 6. Discussion

In this section, we discuss the results of our experiments with MQCC and compare them against the other models in terms of the number of required training parameters, the accuracy of the model, and the circuit depth of the implemented model. The number of qubits required by MQCC can be easily calculated using (23). The number of qubits required for data encoding and filter implementation can be obtained from the dimensions of the data and filter respectively, i.e., $n=\left\lceil \log_{2}128\right\rceil +\left\lceil \log_{2}128\right\rceil +\left\lceil \log_{2}3\right\rceil =16$ for (128×128×3) data, $n_{f}=\left\lceil \log_{2}4\right\rceil =2$ qubits for four features. Similarly, the number of qubits required for feature classes can be calculated as, for example, $n_{c}=\left\lceil \log_{2}2\right\rceil =1$ for 2 classes. All together, for input data encoded into *n* qubits, the optimized MQCC requires $n+n_{f}+n_{c}=19$ qubits.

### 6.1. Number of Parameters

**Figure 22:**
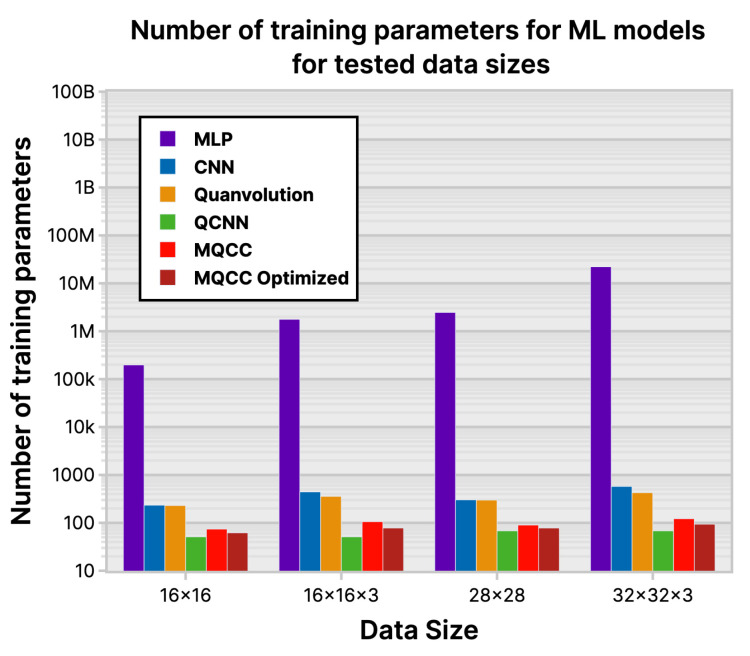
Number of training parameters for ML models for tested data sizes.

Among the classical ML models evaluated, MQCC had the fewest trainable parameters; see Figure 22. This implies potential advantages such as reduced memory usage and faster performance when using classical gradient descent. Although the reduction in parameter decreases from (MLP to CNN) and then further from (CNN to MQCC), parameter reduction diminishes from (MLP to CNN) and further from (CNN to MQCC), and there is still a significant 83.62% decrease in parameter count.

### 6.2. Loss History and Accuracy

**Figure 23:**
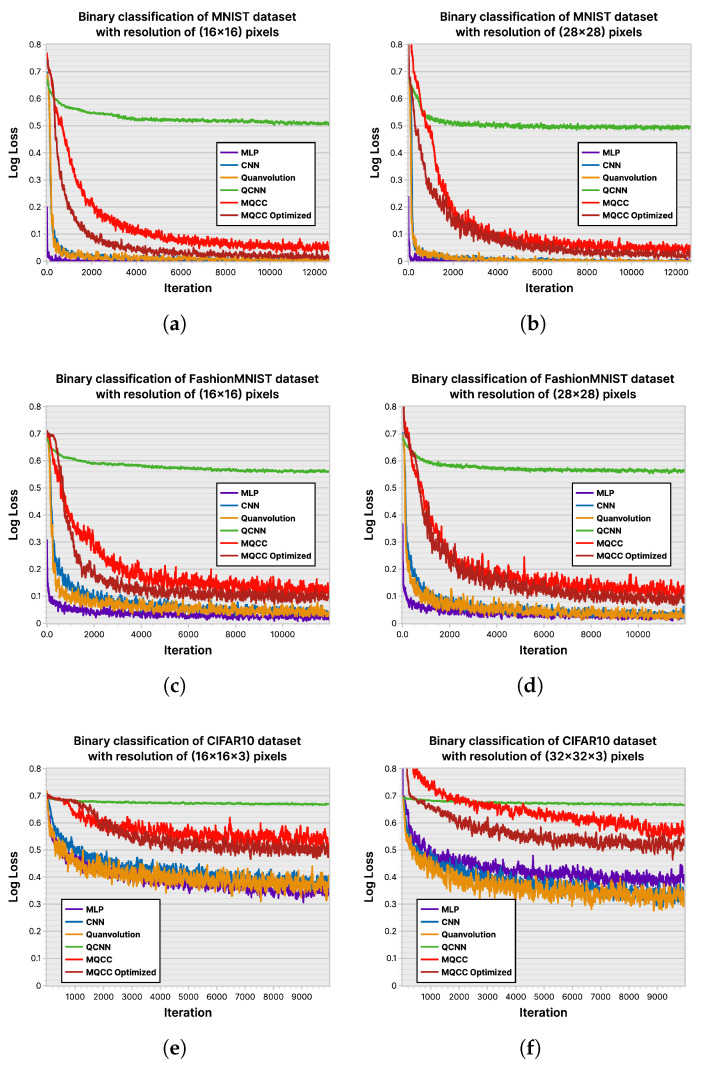
Loss history of ML models on various datasets: (**a**) MNIST (16×16); (**b**) MNIST (28×28); (**c**) FashionMNIST (16×16); (**d**) FashionMNIST (28×28); (**e**) CIFAR10 (16×16×3); (**f**) CIFAR10 (32×32×3).

**Figure 24:**
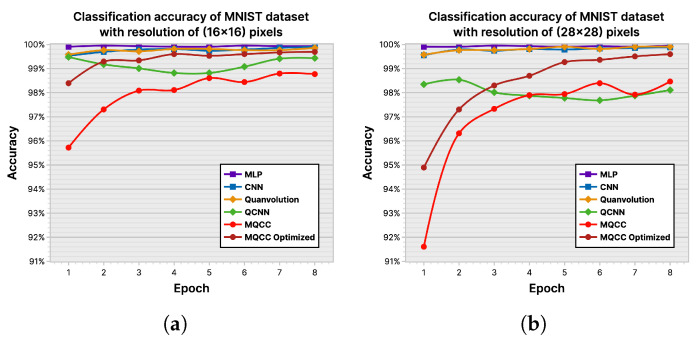

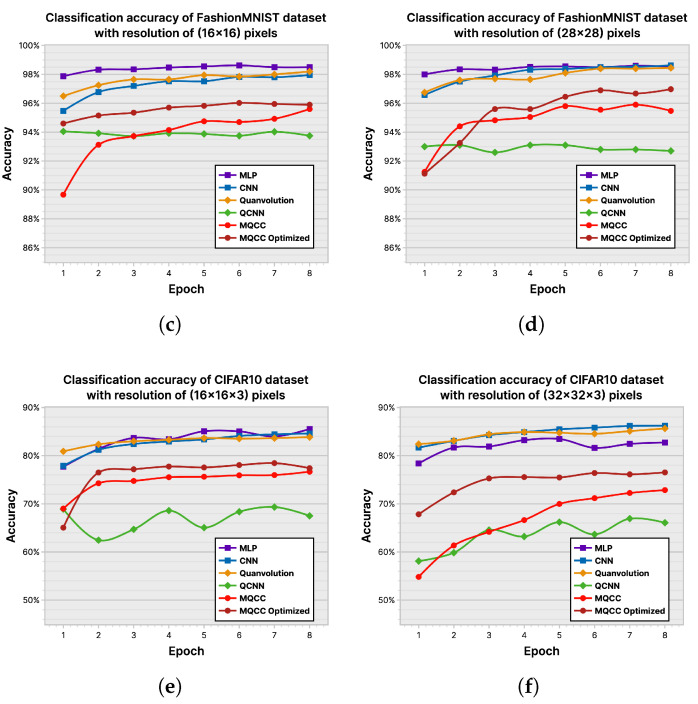
Classification accuracy of ML models on various datasets: (**a**) MNIST (16×16); (**b**) MNIST (28×28); (**c**) FashionMNIST (16×16); (**d**) FashionMNIST (28×28); (**e**) CIFAR10 (16×16×3); (**f**) CIFAR10 (32×32×3).

ML-based classifiers aim to maximize the accuracy of their classifications, measured by a loss function during training to estimate the accuracy that may be exhibited when deployed. Hence, Figure 23 and Figure 24 depict the performance of the ML models across the experimental datasets in their plotting of log-loss history and classification accuracy. The MNIST [42] dataset is not complex enough to effectively distinguish models; however, differences begin to emerge in the FashionMNIST [43] and CIFAR10 [44] datasets. MLP consistently achieves the highest accuracy across trials due to its larger parameter count, allowing for greater flexibility in adapting to nuances in input. The CNN showcases its ability to select relevant input features using convolution and data locality, demonstrating the second-highest accuracy. Among the tested models, QCNN generally performs the poorest, displaying its inability to properly leverage data locality. However, comparing the accuracy of MQCC and quanvolutional neural networks is inconclusive. Quanvolutional neural networks performed better on FashionMNIST, whereas MQCC performed better on CIFAR10.

### 6.3. Gate Count and Circuit Depth

**Figure 25:**
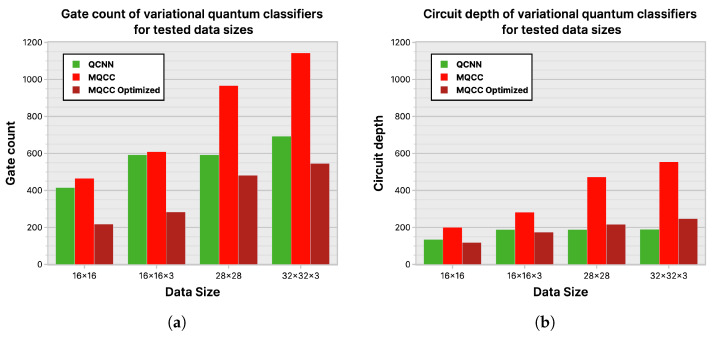
Gate count and circuit depth of MQCC vs. QCNN: (**a**) gate count; (**b**) circuit depth.

Although comparing MQCC, MQCC Optimized, and QCNN with quantum metrics like gate count and circuit depth is viable, see Figure 25, it is challenging to include quanvolutional neural networks in the comparison due to their significant differences from the other models. These differences are due to the quantum component within quanvolutional neural networks constituting a small fraction of the entire algorithm, bringing it closer to a classical algorithm than a quantum algorithm. Meanwhile, comparing the techniques of MQCC and QCNN in Figure 25 highlights the rationale behind developing MQCC Optimized. Initially, MQCC performed worse than QCNN in gate count and circuit depth. However, after optimizations, MQCC matched the performance of QCNN and even outperformed it in the best-case scenarios. While the QCNN architecture appears more suitable for shallower quantum circuits than MQCC, it is because the high parallelization of each QCNN layer halves the active qubits.

Despite QCNN using half the active qubits per layer than MQCC, MQCC utilizes the extra qubits for weights and features, with each pooling layer reducing the qubit count by a constant amount, $n_{k}$. However, as QCNN structures are motivated by the classical convolution operation, they usually need more complex and deeper “convolution” and “pooling” ansatz to attain a higher accuracy.

### 6.4. Complexity Comparison with Classical Models

The proposed method can also be compared with the classical models in terms of temporal complexity. The temporal/depth complexity of MQCC Optimized can be easily derived using Figure 11 and Figure 12, as well as (24), as shown in (35), where $O_{stride}$ and $O_{U_{k}}$ are combined to obtain $O_{conv}$.

(35) $$\begin{array}{cc} O_{MQCC}^{opt}\left(n,n_{k},n_{f},n_{c}\right) & =O_{stride}\left(n-\ell ,n_{k}\right)+O_{U_{k}}\left(n_{k},n_{f}\right)+O_{FC}\left(n-\lambda \ell ,n_{c}\right)\\ \; & =O_{conv}\left(n,n_{k},n_{f}\right)+O_{FC}\left(n-\lambda \ell ,n_{c}\right) \end{array}$$

For a MAC-based fully connected layer, we can consider $U_{\mathrm{FC}}=U_{\mathrm{MAC}}$ to obtain the complexity using (13) and (35), as shown in (36). Similarly, for an ansatz-based fully connected layer, we can consider $U_{\mathrm{FC}}=U_{\mathrm{ansatz}}$ to obtain the complexity using (25) and (35), as shown in (37).

(36) $$\begin{array}{cc} O_{MQCC_{MAC}}^{opt}\; & =O\left(n_{k}n^{2}-n_{k}^{2}n+2^{n_{k}+n_{f}}\right)+O\left(2^{n+n_{c}-\lambda \ell }\right)\\ \; & =O\left(n^{2}\right)+O\left(2^{n}\right)=O\left(2^{n}\right)=O\left(N\right)\approx O_{FC} \end{array}$$

(37) $$\begin{array}{cc} O_{MQCC_{ansatz}}^{opt} & =O\left(n_{k}n^{2}-n_{k}^{2}n+2^{n_{k}+n_{f}}\right)+O\left(n\right)\\ \; & =O\left(n^{2}\right)+O\left(n\right)=O\left({\left(\log_{2}N\right)}^{2}\right)\approx O_{conv} \end{array}$$

The general expression for calculating the temporal complexity of classical CNNs is shown in (38). It can be broadly divided into three parts: the complexity of the convolutional layers, the complexity of the pooling layers, and the complexity of the fully connected layers. The complexity of the combined layers of convolution and pooling is primarily determined by the number of filters, the size of the filters, and the dimensions of the input feature maps, while the complexity of fully connected layers depends on the number of neurons in the layers. It is worth mentioning that in classical CNNs, convolutional layers dominate both the pooling and fully connected layers in the overall execution time, as expressed by (38).

(38) $$\begin{array}{cc} O_{CNN}^{Classical} & =O_{conv}+O_{pooling}+O_{fully\_connected\_layer}\approx O_{conv} \end{array}$$

A comparison of the depth/temporal complexity of MQCC Optimized against the classical method is shown in Table 5. It details the depth complexities of C2Q data encoding (I/O overhead), two variants of MQCC Optimized, i.e., MAC-based fully connected layer and ansatz-based fully connected layer, and three variants of classical CNN implementation. Among the classical CNN variants, i.e., Direct (CPU), FFT (CPU/GPU), and GEMM (GPU), we consider the Direct (CPU) case as the worst case and GEMM (GPU) as the best case. The MQCC-optimized algorithm with a MAC-based fully connected layer matches the best case. Considering the depth of the I/O circuit, which, in this case, is the depth of the C2Q method, the overall complexity of MQCC is still better than the worst case in classical methods. The MQCC-optimized algorithm, with an ansatz-based fully connected layer, has the least complexity among all the compared models. Although the I/O overhead represents the worst-case scenario for our proposed technique (see Table 5), the complexity of our proposed MQCC technique, including the I/O overhead, matches the best case in classical methods. It is worth emphasizing that the I/O overhead is not intrinsic to our proposed technique; rather, it is a general consideration for any data-intensive quantum application like quantum machine learning (QML) classification. Moreover, our proposed MQCC method provides two additional advantages over classical CNNs, being highly parallelizable and requiring fewer training parameters, see Figure 22, which ultimately leads to fewer resource requirements than classical CNNs.

## 7. Conclusions

In this paper, we presented a multidimensional quantum convolutional classifier (MQCC) that consists of quantum convolution, quantum pooling, and a quantum fully connected layer. We leveraged existing convolution techniques to support multiple features/kernels and utilized them in our proposed method. Furthermore, we proposed a novel width-optimized quantum circuit that reuses freed-up qubits from the pooling layer in the subsequent convolutional layer. The proposed MQCC additionally preserves data locality in the input data which has shown to improve data classification in convolutional classifiers. The MQCC methodology is generalizable for any arbitrary multidimensional filter operation and pertinent for multifeature extraction. The proposed method can also support data of arbitrary dimensionality since the underlying quantum pooling and convolution operations are generalizable across data dimensions. We experimentally evaluated the proposed MQCC on various real-world multidimensional images, utilizing several filters through simulations on state-of-the-art quantum simulators from IBM Quantum and Xanadu. In our experiments, MQCC achieved higher classification accuracy over contemporary QML methods while having a reduced circuit depth and gate count. In our future work, we are planning on expanding MQCC with additional convolution capabilities, such as arbitrary striding and dilation, and further optimizing it for deployment on real-world quantum processors. In addition, we will investigate using our proposed quantum techniques for real-life applications such as medical imaging and classification.

## Figures and Tables

**Table 1:** The 1D convolution (averaging) filters applied to 1D audio samples [39].

Data Size (No. of Sample Points)	(3) Averaging Classical/ Noise-Free	(3) Averaging Noisy ($10^{6}$ Shots)	(5) Averaging Classical/ Noise-Free	(5) Averaging Noisy ($10^{6}$ Shots)
256	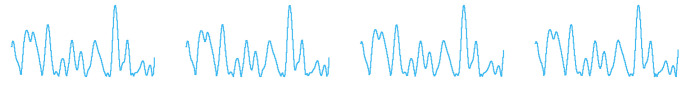			
4096	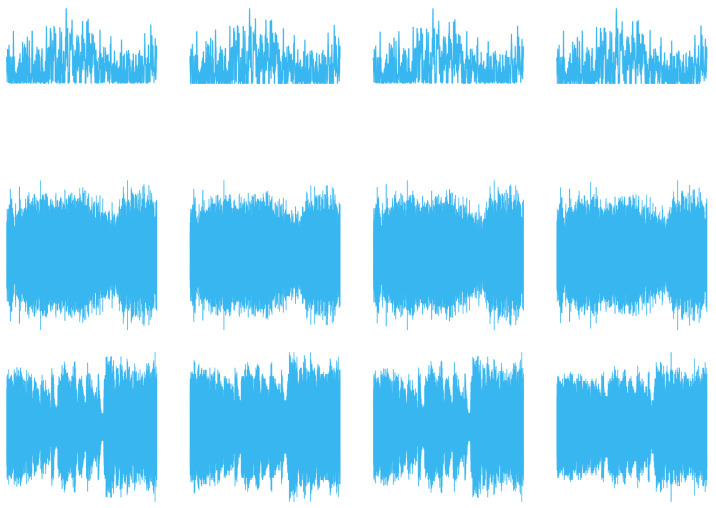			
65,536				
1,048,576				

**Table 2:** Two-dimensional convolution kernels applied to a (128×128) bw image [40].

Kernel	(3×3) Kernel Classical/Noise-Free	(3×3) Kernel Noisy ($10^{6}$ Shots)	(5×5) Kernel Classical/Noise-Free	(5×5) Kernel Noisy ($10^{6}$ Shots)
Average	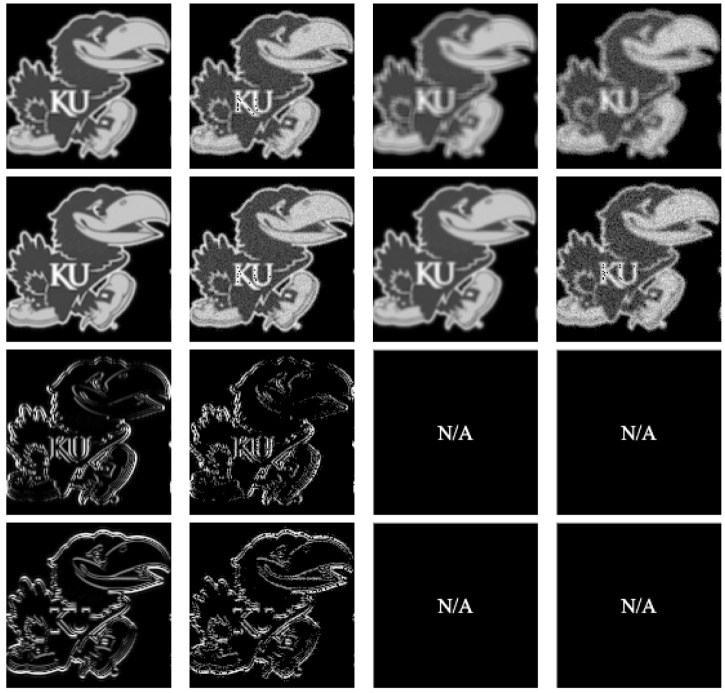			
Gaussian				
Sobel-X				
Sobel-Y				
Laplacian	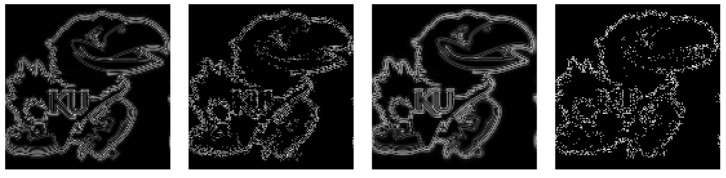			

**Table 3:** Two-dimensional convolution kernels applied to a (128×128×3) rgb image [40].

Kernel	(3×3) Kernel Classical/Noise-Free	(3×3) Kernel Noisy ($10^{6}$ Shots)	(5×5) Kernel Classical/Noise-Free	(5×5) Kernel Noisy ($10^{6}$ Shots)
Average	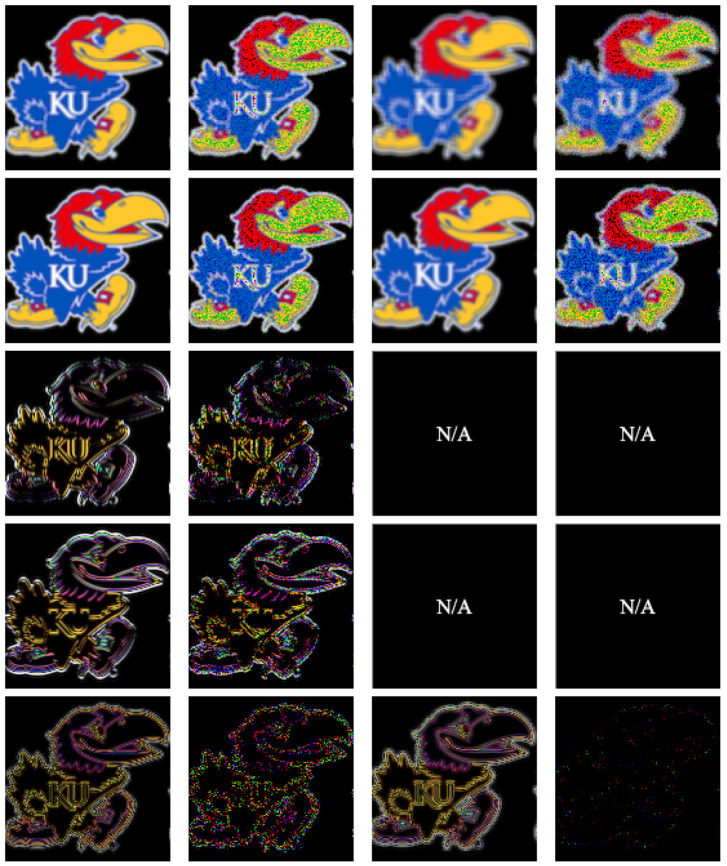			
Gaussian				
Sobel-X				
Sobel-Y				
Laplacian				

**Table 4:** The 3D convolution (averaging) filters applied to 3D hyperspectral images (bands 0, 1, and 2) [41].

Data Size ($N_{\mathrm{rows}}\times N_{\mathrm{cols}}\times N_{\mathrm{bands}}$)	(3×3×3) Averaging Classical/ Noise-Free	(3×3×3) Averaging Noisy ($10^{6}$ Shots)	(5×5×5) Averaging Classical/ Noise-Free	(5×5×5) Averaging Noisy ($10^{6}$ Shots)
(8×8×8)	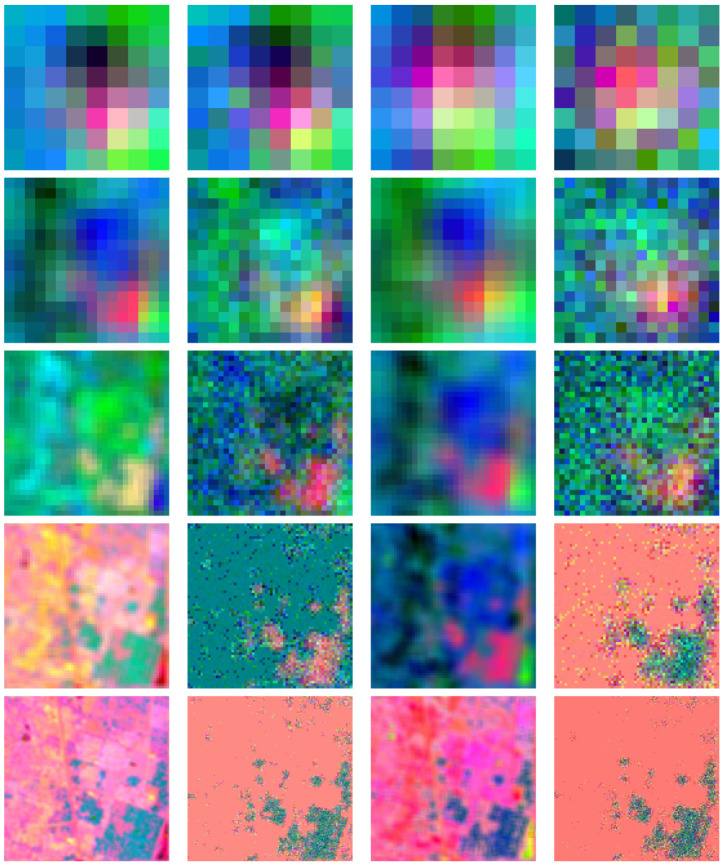			
(16×16×16)				
(32×32×32)				
(64×64×64)				
(128×128×128)				

**Table 5:** Comparison of depth/time complexity of proposed MQCC-optimized against classical CNN.

a Depth complexity of C2Q [12] data encoding (I/O) technique				
	$O_{I/O}\left(2^{n}\right)=O_{I/O}\left(N\right)$			
**b** Complexity of proposed technique compared with classical convolutional neural network				
MQCC Optimized with MAC-based fully connected layer	MQCC Optimized with ansatz-based fully connected layer	Direct (CPU) [21]	FFT (CPU/GPU) [21,22]	GEMM (GPU) [23,24]
$\begin{array}{c} O_{\mathrm{alg}}\left(2^{n})\right.\equiv O_{\mathrm{alg}}\left(N\right)\\ O_{\mathrm{alg}}\;+\;I/O\left(2^{n}+2^{n})\right.\equiv \\ O_{\mathrm{alg}}\;+\;I/O\left(N\right) \end{array}$	$\begin{array}{c} O_{\mathrm{alg}}\left(n^{2})\right.\equiv O_{\mathrm{alg}}\left({\left(\log N\right)}^{2}\right)\\ O_{\mathrm{alg}}\;+\;I/O\left(n^{2}+2^{n})\right.\equiv \\ O_{\mathrm{alg}}\;+\;I/O\left(N\right) \end{array}$	$\begin{array}{c} O_{\mathrm{alg}}\left(4^{n}\right)\equiv \\ O_{\mathrm{alg}}\left(N^{2}\right) \end{array}$	$\begin{array}{c} O_{\mathrm{alg}}\left(n2^{n}\right)\equiv \\ O_{\mathrm{alg}}\left(N\log N\right) \end{array}$	$\begin{array}{c} O_{\mathrm{alg}}\left(2^{\left(n+n_{k}\right)}\right)\equiv \\ O_{\mathrm{alg}}\left(N_{K}N\right) \end{array}$

**Figure A1:**
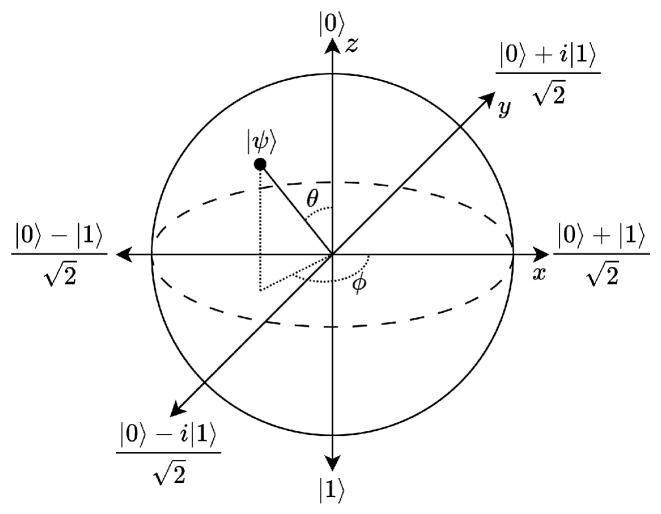
The Bloch sphere.

**Figure A2:**
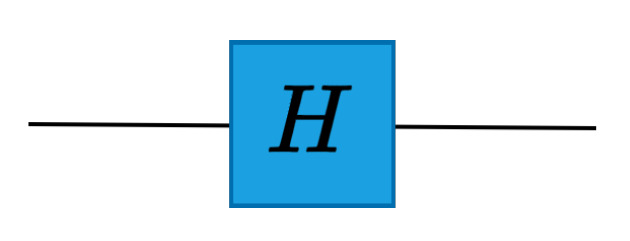
Hadamard gate diagram.

**Figure A3:**
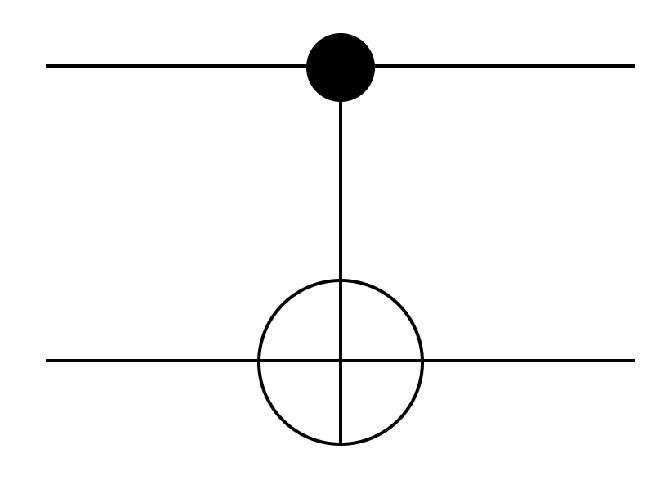
Controlled-NOT gate diagram.

**Figure A4:**
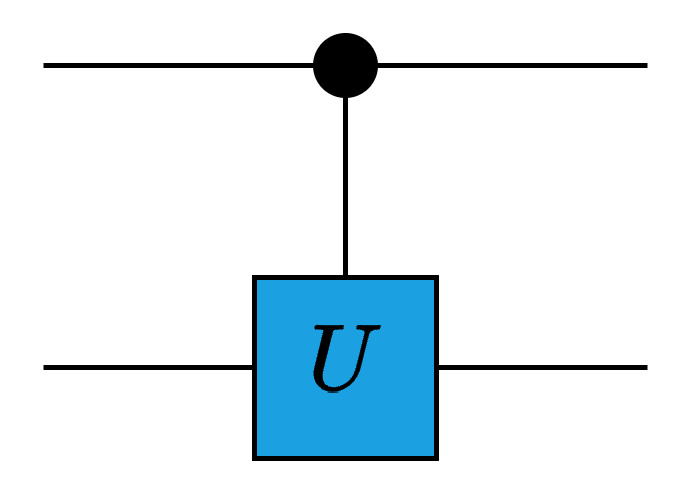
Controlled-U gate diagram.

**Figure A5:**
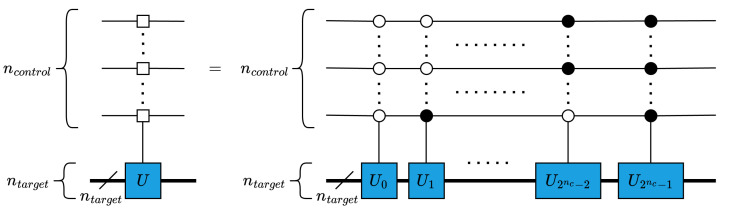
The square box notation for a quantum multiplexer [33].

**Figure A6:**
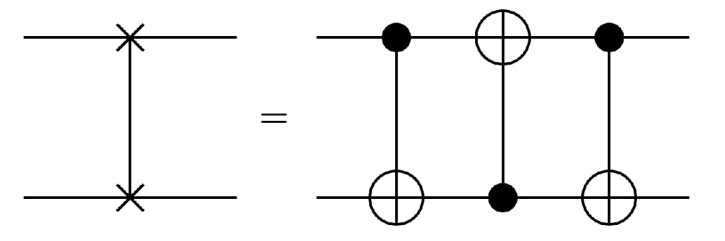
Swap gate diagram and decomposition.

**Figure A7:**
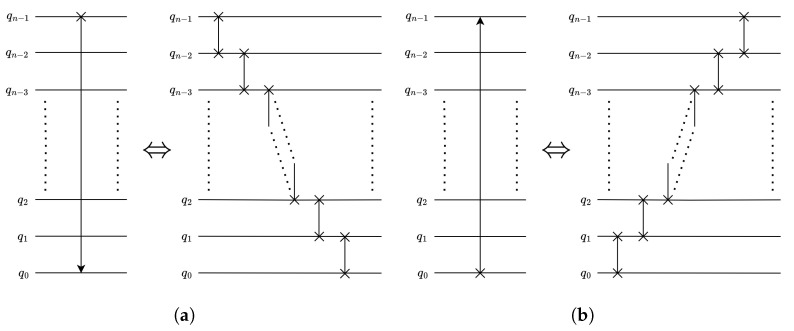
Quantum Perfect-Shuffle Permutations [11]. (**a**) RoL: $|q_{n-1}q_{n-2}\ldots q_{0}\rangle \to |q_{n-2}\ldots q_{0}q_{n-1}\rangle$; (**b**) RoR: $|q_{n-1}\ldots q_{1}q_{0}\rangle \to |q_{0}q_{n-1}\ldots q_{1}\rangle$.

## Data Availability

The audio samples used in this work are publicly available from the European Broadcasting Union at https://tech.ebu.ch/publications/sqamcd (accessed on 23 February 2024) as file 64.flac [39]. The hyperspectral data used in this work are publicly available from the Grupo de Inteligencia Computacional (GIC) at https://www.ehu.eus/ccwintco/index.php/Hyperspectral_Remote_Sensing_Scenes#Kennedy_Space_Center_(KSC) (accessed on 23 February 2024) under the heading Kennedy Space Center (KSC) [41].

## Appendix A

In this section, we briefly discuss fundamental quantum topics including various quantum operations that are relevant to the proposed MQCC. These operations are generally applicable to quantum computing and quantum machine learning.

### Appendix A.1. Quantum Bits and States

A single quantum bit, or qubit, can be represented by a normalized vector |ψ〉 with $N=2^{1}$ elements, called a state vector; see (A1). The state of a qubit is represented as a point on the surface of the Bloch sphere [51], as shown in Figure A1. To preserve the normalization property of a state vector, a qubit’s state can only be expressed as a point on the sphere’s surface [10].

(A1) $$|\psi \rangle =c_{0}|0\rangle +c_{1}|1\rangle =\left[\begin{array}{c} c_{0}\\ c_{1} \end{array}\right],\;\mathrm{where}\;c_{0}^{2}+c_{1}^{2}=1$$

For *n* qubits, the state vector |ψ〉 has $N=2^{n}$ elements. As shown in (A2), each element $c_{j}\in C$ of |ψ〉 represents the amplitude/coefficient of the $j^{th}$ entry in |ψ〉, or the basis state |*j*〉 [10].

(A2) $$|\psi \rangle =\sum_{j=0}^{N-1}c_{j}\cdot |j\rangle =\left[\begin{array}{c} c_{0}\\ c_{1}\\ \vdots \\ c_{j}\\ \vdots \\ c_{N-2}\\ c_{N-1} \end{array}\right],\;\mathrm{where}\;\sum_{j=0}^{N-1}|c_{j}{|}^{2}=1\;,\;\mathrm{and}\;0\leq j<\left(N=2^{n}\right)$$

### Appendix A.2. Rotation Gates

The rotation gates, $R_{x}\left(\theta \right)$, $R_{y}\left(\theta \right)$, and $R_{z}\left(\phi \right)$, are parameterized versions of the Pauli single-qubit gates, shown in (A3), (A4) and (A5), respectively [33]. In QML, rotation gate parameters are trained to produce better models [5,7].

(A3) $$R_{x}\left(\theta \right)=\left[\begin{array}{cc} \cos(\frac{\theta }{2}) & -i\sin(\frac{\theta }{2})\\ -i\sin(\frac{\theta }{2}) & \cos(\frac{\theta }{2}) \end{array}\right]$$

(A4) $$R_{y}\left(\theta \right)=\left[\begin{array}{cc} \cos(\frac{\theta }{2}) & -\sin(\frac{\theta }{2})\\ \sin(\frac{\theta }{2}) & \cos(\frac{\theta }{2}) \end{array}\right]$$

(A5) $$R_{z}\left(\phi \right)=\left[\begin{array}{cc} e^{-\frac{i\phi }{2}} & 0\\ 0 & e^{\frac{i\phi }{2}} \end{array}\right]$$

### Appendix A.3. Hadamard Gate

The Hadamard gate is a fundamental quantum gate that puts the qubit into a superposition of two basis states, the |0〉 and |1〉 states; see (A6) and Figure A2 [10]. Similarly, by applying Hadamard gates on *n* qubits independently, an *n*-qubit superposition can be created that comprises of $2^{n}$ basis states, as shown in (A7).

(A6) $$H=\frac{1}{\sqrt{2}}[\begin{array}{cc} 1 & 1\\ 1 & -1 \end{array}]$$

(A7)
$$H^{⊗n}\cdot {|0\rangle }^{⊗n}=\frac{1}{\sqrt{2^{n}}}\sum_{j=0}^{2^{n}-1}|j\rangle$$

### Appendix A.4. Controlled-NOT (CNOT) Gate

CNOT gates can form a fundamental set of basis gates in conjunction with single-qubit rotation gates [33]; see (A8) and Figure A3. CNOT gates can be used to entangle two qubits, where a measurement of one qubit can provide information about the second qubit [33].

(A8) $$\mathrm{CNOT}=\mathrm{cX}=(|0\rangle \langle 0|⊗I)+(|1\rangle \langle 1|⊗X)=\left[\begin{array}{cccc} 1 & 0 & 0 & 0\\ 0 & 1 & 0 & 0\\ 0 & 0 & 0 & 1\\ 0 & 0 & 1 & 0 \end{array}\right]$$

All gates, single- or multiqubit, can be extended to have control qubit(s), as shown for a general U in (A9) and Figure A4 [33].

(A9) $$\mathrm{cU}=(|0\rangle \langle 0|⊗I)+(|1\rangle \langle 1|⊗U)=\left[\begin{array}{cc} I & 0\\ 0 & U \end{array}\right]$$

When a general multiqubit gate is extended to have more control qubits, it becomes an *n*-qubit multiple-controlled U (MCU) gate. In the context of CNOT gates, the operation becomes a multiple-controlled-not (MCX) gate [52]. For an *n*-qubit MCX gate with n−1 control qubits and 1 target qubit (see (A10)), the depth of an MCX gate with the addition of a single extra qubit can be found in (A11) [52].

(A10) $$\mathrm{MCX}\left(n\right)=\sum_{i=0}^{2^{n-1}-2}(|i\rangle \langle i|⊗I)+(|2^{n-1}-1\rangle \langle 2^{n-1}-1|⊗X)$$

(A11) $$\Delta_{\mathrm{MCX}}\left(n\right)\leq 48n-196=O\left(n\right)\;\mathrm{for}\;\mathrm{large}\;n$$

The most general controlled gate is the multiplexer, see (A12), which defines a quantum operation to be applied on $n_{target}$ qubits for each state permutation of some control qubit(s), $n_{control}$ [33]. In quantum circuits, the square box notation is used to denote a multiplexer operation, as shown in Figure A5 [33].

(A12) Umux=∑i=02nc−1|i〉〈i|⊗Ui=U0⋱U2ncontrol−1↕2ntarget⋮↕2ntarget0⋮02ntarget+ncontrol

Here, $U_{i}$ is a matrix of size $2^{n_{target}}\times 2^{n_{target}}$ defining the unitary operations/gates applied on each data qubit for the corresponding values *i*, where, $0<i<2^{n_{control}-1}$.

### Appendix A.5. SWAP Gate

In a quantum circuit, SWAP gates are applied to interchange the position of two qubits, as shown in (A13) and Figure A6. The SWAP gate can be decomposed into three CNOT gates [10]; see Figure A6.

(A13) $$\mathrm{SWAP}=\left[\begin{array}{cccc} 1 & 0 & 0 & 0\\ 0 & 0 & 1 & 0\\ 0 & 1 & 0 & 0\\ 0 & 0 & 0 & 1 \end{array}\right]$$

### Appendix A.6. Quantum Perfect-Shuffle Permutation (PSP)

The quantum perfect-shuffle permutations (PSPs) are operations that perform a cyclical rotation of given input qubits and can be implemented using SWAP gates. Figure A7 shows the rotate-left (RoL) and rotate-right (RoR) PSP operations [11] with (n−1) SWAP operations, or 3(n−1) equivalent CNOT operations. More information about CNOT and SWAP gates can be found in Appendix A.4 and Appendix A.5, respectively.

### Appendix A.7. Quantum Shift Operation

The quantum shift operation also performs a cyclic rotation of the basis states [20] similar to the quantum perfect-shuffle operations. Here, the basis states of the *n*-qubit state |ψ〉 are shifted by +k or −k positions by applying a shift operation $U_{\mathrm{shift}}^{\pm k}$; see (A14). Here, the value of *k* determines the type of operation such that when k>0, and the corresponding shift operation is denoted as a quantum incrementer, as shown in Figure A8a [30,53], and when k<0, the corresponding shift operation is denoted as a quantum decrementer [30,53], as shown in Figure A8b. The quantum shift operation plays a vital role in convolution operation by striding the filter operation over data windows [25].

(A14) $$\begin{array}{cc} U_{\mathrm{shift}}^{\pm k}|\psi \rangle & =\sum_{i=0}^{2^{n}-1}c_{i}|j\rangle ,\;\mathrm{where}\;j=\left(i\pm k\right)\;\mathrm{mod}\;2^{n}\\ \; & =\left(\prod_{i=0}^{k-1}U_{\mathrm{shift}}^{\pm 1}\right)|\psi \rangle ={\left(U_{\mathrm{shift}}^{\pm 1}\right)}^{k}|\psi \rangle \end{array}$$

Each quantum shift operation $U_{\mathrm{shift}}^{\pm 1}$ acting on an *n*-qubit state can be decomposed into a pyramidal structure of *n*× MCX gates in a series pattern [20], as shown in Figure A8a,b. In terms of fundamental single-qubit and CNOT gates, the depth of a quantum shift operation with ±1 can be demonstrated as quadratic [52]; see (A15). Generalized quantum shift operations $U_{\mathrm{shift}}^{\pm k}$ can be derived using $k\times U_{\mathrm{shift}}^{\pm 1}$ operations, from the expression in (A14), and the corresponding circuit depth can be derived as shown in (A16).

(A15) $$\begin{array}{c} \begin{array}{cc} \Delta_{U_{\mathrm{shift}}^{-1}}\left(n\right)\; & =\sum_{i=0}^{n-1}\Delta_{\mathrm{MCX}}\left(i\right)\\ \; & =\sum_{i=0}^{n-1}48i-196\\ \; & \leq 24n^{2}-220n=O\left(n^{2}\right)\;\mathrm{for}\;\mathrm{large}\;n \end{array} \end{array}$$

(A16) $$\begin{array}{c} \begin{array}{cc} \Delta_{U_{\mathrm{shift}}^{\pm k}}\left(n,k\right) & =k\cdot \Delta_{U_{\mathrm{shift}}^{\pm 1}}\left(n\right)=O\left(kn^{2}\right) \end{array} \end{array}$$

Now, $U_{\mathrm{shift}}^{\pm k}$ can be expressed as a sequence of controlled shift operations, as shown in (A17). Such operations can be denoted as $U_{\mathrm{shift}}^{\pm k_{j}2^{j}}\left(n\right)$, where 0≤j<n and (n) indicates that the shift operation is applied to an *n*-qubit state, as shown in (A18). Instead of applying sequential $U_{\mathrm{shift}}^{\pm 1}\left(n\right)$ operations, each $U_{\mathrm{shift}}^{\pm 2^{j}}\left(n\right)$ operation can be performed using a single $U_{\mathrm{shift}}^{\pm 1}\left(n-j\right)$ operation; see (A18). Therefore, a more depth-efficient decomposition is shown in Figure A8, and the corresponding depth is provided in (A19).

(A17) $$k={\left(k_{n-1}k_{n-2}\cdots k_{j}\cdots k_{1}k_{0}\right)}_{2}=\sum_{j=0}^{n-1}k_{j}2^{j}:k_{j}\in \left\{0,1\right\}$$

(A18) $$\begin{array}{cc} U_{\mathrm{shift}}^{\pm k}\left(n\right) & =U_{\mathrm{shift}}^{\pm \sum_{j=n-1}^{0}k_{j}2^{j}}\left(n\right)=\prod_{j=n-1}^{0}U_{\mathrm{shift}}^{\pm k_{j}2^{j}}\left(n\right)\\ \; & =\prod_{j=n-1}^{0}\left(U_{\mathrm{shift}}^{\pm k_{j}}\left(n-j\right)⊗I^{⊗j}\right) \end{array}$$

(A19) $$\begin{array}{cc} \Delta_{U_{\mathrm{shift}}^{\pm k}}^{\mathrm{opt}}\left(n\right) & \leq \sum_{j=0}^{n-1}\Delta_{U_{\mathrm{shift}}^{-1}}\left(n-j\right)=24\sum_{j=0}^{n-1}j^{2}-220\sum_{j=0}^{n-1}j\\ \; & \leq 8n^{3}-122n^{2}+114n=O\left(n^{3}\right)\;\mathrm{for}\;\mathrm{large}\;n \end{array}$$
